# The root zone of graminoids: A niche for H_2_-consuming acetogens in a minerotrophic peatland

**DOI:** 10.3389/fmicb.2022.978296

**Published:** 2022-08-05

**Authors:** Anja B. Meier, Sindy Oppermann, Harold L. Drake, Oliver Schmidt

**Affiliations:** ^1^Department of Ecological Microbiology, University of Bayreuth, Bayreuth, Germany; ^2^Department of Arctic and Marine Biology, UiT The Arctic University of Norway, Tromsø, Norway

**Keywords:** anaerobes, acetogenesis, methanogensis, peatlands, microbiome, wetland plant roots, clostridiaceae, holophagaceae

## Abstract

The importance of acetogens for H_2_ turnover and overall anaerobic degradation in peatlands remains elusive. In the well-studied minerotrophic peatland fen Schlöppnerbrunnen, H_2_-consuming acetogens are conceptualized to be largely outcompeted by iron reducers, sulfate reducers, and hydrogenotrophic methanogens in bulk peat soil. However, in root zones of graminoids, fermenters thriving on rhizodeposits and root litter might temporarily provide sufficient H_2_ for acetogens. In the present study, root-free peat soils from around the roots of *Molinia caerulea* and *Carex rostrata* (i.e., two graminoids common in fen Schlöpnnerbrunnen) were anoxically incubated with or without supplemental H_2_ to simulate conditions of high and low H_2_ availability in the fen. In unsupplemented soil treatments, H_2_ concentrations were largely below the detection limit (∼10 ppmV) and possibly too low for acetogens and methanogens, an assumption supported by the finding that neither acetate nor methane substantially accumulated. In the presence of supplemental H_2_, acetate accumulation exceeded CH_4_ accumulation in *Molinia* soil whereas acetate and methane accumulated equally in *Carex* soil. However, reductant recoveries indicated that initially, additional unknown processes were involved either in H_2_ consumption or the consumption of acetate produced by H_2_-consuming acetogens. 16S rRNA and 16S rRNA gene analyses revealed that potential acetogens (*Clostridium, Holophagaceae*), methanogens (*Methanocellales, Methanobacterium*), iron reducers (*Geobacter*), and physiologically uncharacterized phylotypes (*Acidobacteria, Actinobacteria, Bacteroidetes*) were stimulated by supplemental H_2_ in soil treatments. Phylotypes closely related to clostridial acetogens were also active in soil-free *Molinia* and *Carex* root treatments with or without supplemental H_2_. Due to pronounced fermentation activities, H_2_ consumption was less obvious in root treatments, and acetogens likely thrived on root organic carbon and fermentation products (e.g., ethanol) in addition to H_2_. Collectively, the data highlighted that in fen Schlöppnerbrunnen, acetogens are associated to graminoid roots and inhabit the peat soil around the roots, where they have to compete for H_2_ with methanogens and iron reducers. Furthermore, the study underscored that the metabolically flexible acetogens do not rely on H_2_, potentially a key advantage over other H_2_ consumers under the highly dynamic conditions characteristic for the root-zones of graminoids in peatlands.

## Introduction

Acetogens are a polyphyletic group of anaerobes that can use the reductive acetyl-CoA pathway for dissimilation (Drake et al., 2008; Ragsdale, 2008). During hydrogenotrophic acetogenesis H_2_ is used as electron donor (4H_2_ + CO_2_ → CH_3_COO^–^ + H^+^) whereas organic electron donors (e.g., glucose [C_6_H_12_O_6_ → 3CH_3_COO^–^ + 3H^+^] or ethanol [2CH_3_CH_2_OH + 2CO_2_ → 3CH_3_COO^–^ + 3H^+^]) are used during organotrophic acetogenesis (Lever, 2012; Schuchmann and Müller, 2014, 2016). Considering their metabolic versatility, it is not surprising that acetogens were isolated from various ecosystems including peatlands (Kotsyurbenko et al., 1995; Simankova et al., 2000; Drake et al., 2006; Gößner et al., 2008). Peatlands are waterlogged soil ecosystems that are of global relevance, especially because of their function as sinks for CO_2_ and sources for CH_4_ (Aselmann and Crutzen, 1989; Yu et al., 2010; Abdalla et al., 2016; Harenda et al., 2018). Despite recent findings showing that acetogens contribute to the production of acetate in some peatlands, their ecological significance in these ecosystems is still conceptualized rather than resolved (Drake et al., 2009; Hunger et al., 2011; Hädrich et al., 2012; Ye et al., 2014; Kotsyurbenko et al., 2019).

The marked accumulation of acetate and increasing relative abundances of acetogenic taxa in peat soil incubations with supplemental H_2_ suggested that acetogens can successfully compete for H_2_ when it is available at sufficiently high concentrations in peatlands (Kotsyurbenko et al., 1996; Bräuer et al., 2004; Wüst et al., 2009; Hädrich et al., 2012; Hunger et al., 2015). However, at the low H_2_ concentrations that are characteristic for bulk peat soil, acetogens are conceptualized to be outcompeted by methanogens and other H_2_ consumers with lower H_2_ thresholds (Drake et al., 2009; Estop-Aragonés et al., 2013; Kotsyurbenko et al., 2019). The competitiveness of acetogens increases at low temperatures predominating in northern peatlands (Conrad and Wetter, 1990; Nozhevnikova et al., 1994; Kotsyurbenko et al., 2001; Metje and Frenzel, 2005, 2007; Lever, 2012). Furthermore, it was suggested that acetogens may thrive in microenvironments within the peat soil in which H_2_ concentrations might be higher than in the surrounding bulk peat (Hädrich et al., 2012; Ye et al., 2014; Schmidt et al., 2016).

The rootzones of graminoids (i.e., grass-like wetland plants) may represent such microenvironments suited for peat acetogens. Graminoids like *Carex rostrata* (bottle sedge; hereafter *Carex*) and *Molinia caerulea* (purple moor grass; hereafter *Molinia*) are common especially in minerotrophic peatlands (i.e., fens; Eurola et al., 1984; Končalová, 1990). Recently, product profiles in *Carex* and *Molinia* root treatments with or without fen soil showed that H_2_, formed during the fermentation of root-derived organic carbon, accumulated to 0.7–4.5 mM (0.8–5.1 kPa); thus, sufficiently high to support hydrogenotrophic acetogenesis (Meier et al., 2021). In another study, formate [or formate-derived H_2_ (i.e., H_2_ released during the oxidation of formate by formate hydrogenlyase-containing taxa)], which is likely released during the fermentative degradation of root exudates (Koelbener et al., 2010), stimulated acetate production in soil-free *Carex* root treatments as well as root-free fen soil treatments, suggesting that acetogens are associated to graminoid roots and inhabit the soil surrounding the roots (Hunger et al., 2016).

Based on the findings of the two earlier studies (Hunger et al., 2016; Meier et al., 2021), roots and soil from the root zones of *Carex* and *Molinia* were incubated separately, and the effects of supplemental H_2_ on the product profiles and prokaryotic communities were evaluated in order to address the following hypotheses: (1) acetogens are associated to the roots of fen graminoids and inhabit the peat soil surrounding these roots; (2) acetogens can thrive on H_2_ derived from the fermentation of root organic carbon; (3) in the absence of root-derived organic carbon, acetogens are outcompeted for endogenous H_2_ by H_2_ consumers with lower thresholds.

## Materials and methods

### Sampling site and setup of anoxic incubations

Fen Schlöppnerbrunnen is a moderately acidic (pH 4.3–5.6), minerotrophic, CH_4_-emitting fen that is completely overgrown with *M. caerulea*, intermingled with patches of sedges (e.g., *C. rostrata*), rushes, and mosses; the fen is located in the Lestenbach catchment in the Fichtelgebirge (50°07′53″N and 11°52′51″E), Germany (Hamberger et al., 2008; Reiche et al., 2009; Hädrich et al., 2012).

The sampling of roots and soil as well as the setup of anoxic incubations largely resembled that of previous studies (Hunger et al., 2016; Meier et al., 2021) and is summarized in Supplementary Figure 1. *Carex* roots and *Carex* soil (i.e., peat soil from around the *Carex* roots) were sampled in July 2016; *Molinia* roots and *Molinia* soil (i.e., peat soil from around the *Molinia* roots) were sampled in July 2018. Samples were transported to the lab in airtight sterile plastic bags on ice and transferred in an anoxic chamber (100% N_2_ atmosphere, Mecaplex, Grenchen, Switzerland). Roots were separated from the soil and washed gently with sterile anoxic water to remove residual soil particles; soil was sieved to obtain soil largely devoid of roots, termed root-free soil. One gram fresh weight of roots or soil were transferred in 27 ml glass tubes and 9 ml of anoxic mineral solution (Hunger et al., 2015) were added to make up a total volume of 10 ml. Tubes were sealed with butyl-rubber stoppers and flushed with 100% N_2_. Approximately 10 μmol H_2_ per ml liquid volume was added to H_2_ treatments, whereas no H_2_ was added to unsupplemented treatments. The following abbreviations are used for H_2_ treatments and unsupplemented treatments: SUC, unsupplemented *Carex* soil; SHC, H_2_ supplemented *Carex* soil; SUM, unsupplemented *Molinia* soil; SHM, H_2_ supplemented *Molinia* soil; RUC, unsupplemented *Carex* roots; RHC, H_2_ supplemented *Carex* roots; RUM, unsupplemented *Molinia* roots; and RHM, H_2_ supplemented *Molinia* roots. All treatments were setup in triplicates and incubated in the dark at 15°C for 17 days (without a pre-incubation).

### Chemical analyses

The headspaces and liquid phases of H_2_ treatments and unsupplemented treatments were sampled regularly during the incubation using sterile syringes. The devices and instrumental setup used for (a) gas chromatographic analysis of headspace gasses, (b) high performance liquid chromatography analysis of organic acids and ethanol, and (c) pH measurements were those recently described in detail (Meier et al., 2021). Amounts of CO_2_ (including pH-dependent amounts of bicarbonate), H_2_, and CH_4_ in the headspaces and liquid phases were calculated as described before (Meier et al., 2021), and molar concentrations of gasses were calculated by dividing total amounts of a gas (in μmol) by 9.5 ml (the volume of the liquid phase after initial sampling). Dry weight contents (determined by weighing before and after drying at 60°C for 72 h) of the roots and soil in the three experiments were as follows: *Carex* soil/roots, 11%/13%; *Molinia* soil/roots, 16%/36%. Millimolar concentrations of gasses, organic acids, and ethanol can be converted to μmol per g dry weight by multiplying with 86/73 for *Carex* soil/roots and 59/26 for *Molinia* soil/roots.

### Reductant recoveries and thermodynamic calculations

Reductant recoveries were calculated to determine whether the enhanced accumulation of acetate and methane in H_2_ supplemented soil treatments compared to unsupplemented soil treatments could be explained by the consumption of exogenous H_2_. Reductant recoveries for acetate (*R*_*A*_) and CH_4_ (*R*_*M*_) were calculated according to the following Equations.


$${\text{R}}_{A}=\frac{8\times \left(\left({\left[\text{Ah}\right]}_{t2}-{\left[\text{Ah}\right]}_{t1}\right)-\left({\left[\text{Au}\right]}_{t2}-{\left[\text{Au}\right]}_{t1}\right)\right)}{2\times \left(\left({\left[\text{Hh}\right]}_{t2}-{\left[\text{Hh}\right]}_{t1}\right)-\left({\left[\text{Hu}\right]}_{t2}-{\left[\text{Hu}\right]}_{t1}\right)\right)}\times -100\%$$

$${\text{R}}_{M}=\frac{8\times \left(\left({\left[\text{Mh}\right]}_{t2}-{\left[\text{Mh}\right]}_{t1}\right)-\left({\left[\text{Mu}\right]}_{t2}-{\left[\text{Mu}\right]}_{t1}\right)\right)}{2\times \left(\left({\left[\text{Hh}\right]}_{t2}-{\left[\text{Hh}\right]}_{t1}\right)-\left({\left[\text{Hu}\right]}_{t2}-{\left[\text{Hu}\right]}_{t1}\right)\right)}\times -100\%$$

In the equations above, eight refers to the number of reducing equivalents per molecule acetate and methane; two refers to the reducing equivalents per molecule H_2_. [Ah], [Au], [Mh], [Mu], [Hh], and [Hu] are the concentrations of acetate in H_2_ supplemented soil treatments, acetate in unsupplemented soil treatments, CH_4_ in H_2_ supplemented soil treatments, CH_4_ in unsupplemented soil treatments, H_2_ in H_2_ supplemented soil treatments, and H_2_ in unsupplemented soil treatments, respectively, at the start (*t*_1_) and the end (*t*_2_) of the respective time frame.

Gibb’s free energies (ΔG) were calculated using the Nernst and Van’t Hoff equations (Conrad and Wetter, 1990).

### Extraction of nucleic acids and reverse transcription of RNA

Three replicate nucleic acid extractions were performed with fresh washed roots and sieved soil to analyze the *in situ* microbial community at the time of sampling. At the end of the 17-day incubation, nucleic acids were extracted from all replicates of root and soil treatments separately. Samples of soil treatments were centrifuged at 16,000 × *g* for 15 min at 4°C (1-15-K Sartorius, Göttingen, Germany) to retrieve pelleted soil suitable for nucleic acid extraction. Nucleic acid extraction, digestion of DNA (to retrieve pure RNA) or RNA (to retrieve pure DNA), and cDNA synthesis were performed as described before (Meier et al., 2021).

### Molecular analyses

PCR amplification, Illumina MiSeq amplicon sequencing, and data processing were performed as stated elsewhere (Zeibich et al., 2019; Meier et al., 2021). In short, primers Pro341f (5′-CCT ACG GGN BGC ASC AG-3′) and Pro805r (5′-GAC TAC NVG GGT ATC TAA TCC-3′; Takahashi et al., 2014) were used for 16S rRNA amplicon generation, quality filtered sequences were clustered using a 97% similarity cut-off, and chloroplast- and mitochondria-related sequences were excluded from further analyses.

### Identification of phylotypes stimulated by supplemental H_2_ in soil treatments

Linear discriminant analysis effect size (LEfSe; Segata et al., 2011) was conducted with 16S rRNA and 16S rRNA gene sequence data separately for *Carex* and *Molinia* soil treatments. Phylotypes that fulfilled the following three criteria based on 16S rRNA or 16S rRNA gene sequence data were designated as “stimulated” by supplemental H_2_: Phylotypes (a) were significantly (*P* ≤ 0.05; Kruskal–Wallis test) more abundant in H_2_ supplemented soil treatments compared to unsupplemented soil treatments, (b) had an effect size (LDA score) of ≥3, and (c) were on average at least twice as abundant in H_2_ supplemented soil treatments as in unsupplemented soil treatments (the proof of criteria c is not implemented in LEfSe and was performed manually in order to eliminate phylotypes that were only slightly more abundant in H_2_ supplemented soil treatments compared to unsupplemented soil treatments).

### Statistical analyses

One-sided Wilcoxon rank sum test implemented in R^1^ was used to identify statistically significant differences (*P* value of ≤ 0.05) between the amounts of CH_4_ and acetate formed in H_2_ treatments and unsupplemented treatments during the 17-day incubation. Differences in the overall composition of prokaryotic communities before incubation and after incubation in H_2_ treatments and unsupplemented treatments were visualized by non-metric multidimensional scaling (NMDS) based on the Bray-Curtis distance matrix calculated using the software Past3^2^ (Hammer et al., 2001).

### Accession numbers

Sequences were deposited at the European Nucleotide Archive under study numbers PRJEB37304 and PRJEB37863 for *Carex* and *Molinia* experiments, respectively. Representative sequences of phylotypes stimulated by supplemental H_2_ in soil treatments were deposited under the accession numbers LR792771-LR792783 and LR792811-LR792818.

## Results and discussion

### Response to H_2_ in soil treatments

In the fen Schlöppnerbrunnen, excess H_2_ formed by root associated fermenters might occasionally diffuse into the peat soil surrounding graminoid roots where it might stimulate H_2_ consumers that relay on H_2_ concentrations higher than the 0.2–28 nmol l^–1^ dissolved H_2_ (corresponds to a H_2_ partial pressure of approximately 0.03–4 Pa) observed in bulk peat soil *in situ* (Knorr et al., 2009; Estop-Aragonés et al., 2013; Hunger et al., 2016; Meier et al., 2021). To simulate contrasting H_2_ availabilities in the peat soil, soil treatments with supplemental H_2_ und unsupplemented soil treatments were conducted.

**FIGURE 1:**
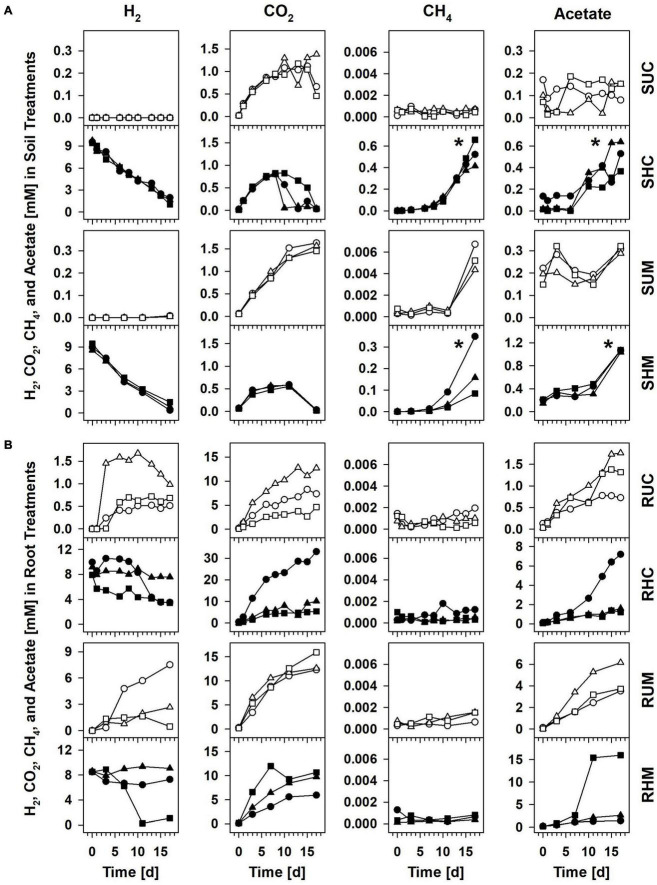
Concentrations of H_2_, CO_2_, CH_4_, and acetate in soil treatments **(A)** and root treatments **(B)**. Treatment identifiers: SUC, unsupplemented *Carex* soil; SHC, H_2_ supplemented *Carex* soil; SUM, unsupplemented *Molinia* soil; SHM, H_2_ supplemented *Molinia* soil; RUC, unsupplemented *Carex* roots; RHC, H_2_ supplemented *Carex* roots; RUM, unsupplemented *Molinia* roots; RHM, H_2_ supplemented *Molinia* roots. Symbols: circles, replicate 1; triangles, replicate 2; squares, replicate 3. The asterisks indicate significant differences (one-sided Wilcoxon rank sum test; *P* ≤ 0.05) between the amounts of CH_4_ and acetate formed in H_2_ treatments and unsupplemented treatments during incubation. See Supplementary Figure 2 for ethanol, butyrate, propionate, and pH.

Supplemental H_2_ was consumed linearly in H_2_ supplemented soil treatments at rates of 0.45 mM H_2_ d^–1^ (*R*^2^ = 0.98) for *Carex* soil (Treatment SHC) and 0.48 mM H_2_ d^–1^ (*R*^2^ = 0.97) for *Molinia* soil (Treatment SHM; Figure 1A). That H_2_ was consumed without delay suggested that H_2_-consuming microbes in the root zones of both plants were poised to respond quickly to the sudden availability of H_2_ at high concentrations. Initially, the consumption of H_2_ was in stark contrast to the accumulation of little acetate and CH_4_ in treatments SHC and SHM (Figure 1A), and reductant recoveries confirmed that both products accounted for only a small fraction of the H_2_ that was consumed within the first 6 to 7 days (Table 1). Thus, neither hydrogenotrophic acetogenesis nor hydrogenotrophic methanogenesis seemed to be main H_2_-consuming processes during the first stage of incubation. With time acetate and CH_4_ accumulation accelerated (Figure 1A), and during the second stage of incubation, both products collectively accounted for 87.4 and 89.6% of consumed exogenous H_2_ in Treatments SHC and SHM, respectively, (Table 1), pointing toward hydrogenotrophic acetogenesis and hydrogenotrophic methanogenesis as the main H_2_-consuming processes between day 6 or 7 and day 17. CO_2_ is the electron acceptor of both processes (Thauer et al., 2008; Schuchmann and Müller, 2014), and its subsequent consumption toward the end of the incubation is in line with ongoing hydrogenotrophic acetogenesis and hydrogenotrophic methanogenesis in Treatments SHC and SHM (Figure 1A). Similar amounts of CH_4_ and acetate were formed in Treatment SHC, whereas at least three times more acetate than CH_4_ accumulated in Treatment SHM (Figure 1A). Small amounts of propionate accumulated toward the end of the incubation in H_2_ supplemented soil treatments (Supplementary Figure 2), a finding in line with the formation of propionate in formate treatments of fen Schlöppnerbrunnen soil (Hunger et al., 2011). In unsupplemented *Molinia* soil (Treatment SUM) low amounts of acetate and CH_4_ accumulated toward the end of the incubation, and in unsupplemented *Carex* soil (Treatment SUC) neither acetate nor CH_4_ accumulated (Figure 1A).

**TABLE 1:** Reductant recoveries for supplemental H_2_ consumed in soil H_2_ treatments ^a^.

Treatment^b^	Incubation period [*d*]	Consumed H_2_ [mM]	% of reductant recovered in a product		
			CH_4_	Acetate	Unknown
SHC	0–6	3.6	2.5	−0.9^c^	98.4
	6–17	4.4	46.3	41.1	12.6
	0–17	8.0	26.5	22.1	51.3
SHM	0–7	4.5	0.6	12.0	87.4
	7–17	3.6	20.6	68.9	10.4
	0–17	8.1	9.5	37.3	53.2

^a^Recoveries were calculated from mean (*n* = 3) concentrations of H_2_, CH_4_, and acetate of H_2_ treatments and unsupplemented treatments (Figure 1A) as described in the material and methods section. Percentages of reductant recovered in unknown products (e.g., sulfide, ferrous iron, and reduced humic acids) were calculated by subtracting percentages of reductant recovered in CH_4_ and acetate from 100%.

^b^Treatments: SHC, H_2_ supplemented *Carex* soil; SHM, H_2_ supplemented *Molinia* soil.

^c^The negative value indicates that initially acetate accumulation was lower in H_2_ supplemented *Carex* soil compared to unsupplemented *Carex* soil.

The collective data suggested that H_2_ consuming acetogens and methanogens inhabiting the peat soil surrounding *Carex* and *Molinia* roots were substrate limited in unsupplemented soil treatments and became increasingly important in H_2_ supplemented soil treatments. While acetogenic and methanogenic potentials were similar in H_2_ treatments with *Carex* soil, acetogenesis was found to exceed methanogenesis in H_2_ treatments with *Molinia* soil. In any way, anaerobic respiratory microbes that use electron acceptors others than CO_2_ were presumably involved in the mineralization of peat organic carbon and in the consumption of supplemental H_2_. It cannot be excluded that such respiratory processes also consumed acetate produced by acetogens, and aceticlastic methanogenesis is another possible sink for acetate.

### Phylotypes stimulated by supplemental H_2_ in soil treatments

Non-metric multidimensional scaling analyses of 16S rRNA and 16S rRNA gene phylotypes (≥97% sequence similarity), alpha diversity parameters, and phylum/family level-based community profiling collectively suggested that supplemental H_2_ had a minor effect on the overall microbial community composition in soil treatments and root treatments of both plants (Supplemental Text 1, Supplementary Figures 3, 4, and Supplementary Tables 1, 2). Thus, a more detailed analysis was necessary to identify potential soil-born or root-associated H_2_ consumers.

**FIGURE 2:**
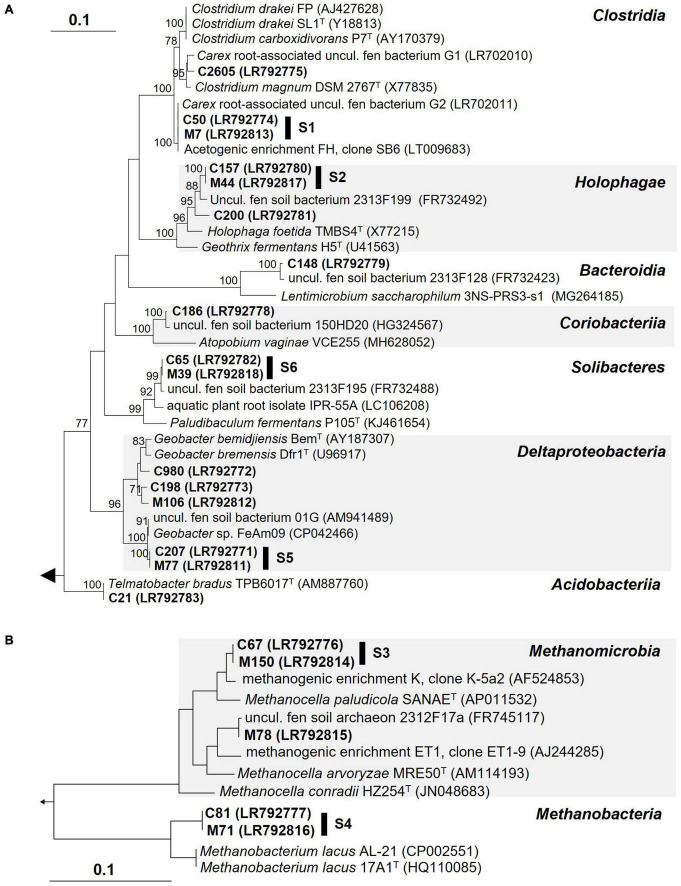
16S rRNA gene-based phylogenetic trees of bacterial **(A)** or archaeal **(B)** phylotypes stimulated by supplemental H_2_ in root-free soil treatments (bold; see Table 2) and related prokaryotes. S, shared phylotypes (i.e., *Carex* phylotypes that shared 100% 16S rRNA gene sequence similarity with *Molinia* phylotypes). The phylogenetic trees were calculated using the neighbor joining function (correction model: Jukes-Cantor) implemented in the ARB software (Ludwig et al., 2004). Bootstrap values (1,000 resamplings) higher than 70% are shown. *Methanosarcina acetivorans* C2A (AE010299) and *Telmatobacter bradus* TPB6017 (AM887760) were used as outgroup in **(A,B)**, respectively.

In order to identify the most important 16S rRNA phylotypes that responded to supplemental H_2_ in soil treatments a two-step approach was conducted (see section “Material and methods” for details). LEfSe analyses (Segata et al., 2011) identified 17 *Carex* phylotypes and 10 *Molinia* phylotypes that (1) were significantly more abundant in H_2_ supplemented soil treatments than in unsupplemented soil treatments and (2) had LDA-scores of three or higher (Table 2). 13 of the 17 *Carex* phylotypes and 6 of the 10 *Molinia* phylotypes were at least twice as abundant in H_2_ supplemented soil treatments than in unsupplemented soil treatments and only these phylotypes were considered as “stimulated by H_2_” (Table 2). Phylogenetic analysis revealed that 6 of the 13 *Carex* phylotypes shared 100% 16S rRNA gene sequence similarity with *Molinia* phylotypes that fulfilled the LEfSe criteria; hereafter, these phylotypes were designated as “shared phylotypes” (S, e.g., phylotype S1 comprises *Carex* phylotype C50 and *Molinia* phylotype M7; Figure 2).

**TABLE 2:** Phylotypes stimulated by supplemental H_2_ in soil treatments ^a^.

PT (S)^b^	LDA-Score^c^		RA ratio	
	16S rRNA^d^	16S rRNA genes^d^	16S rRNA^d^	16S rRNA genes^d^
***Carex* phylotypes**				
*C50 (1)*	3.86^(2)^	3.67^(1)^	21.7	44.3
*C157 (2)*	3.67^(4)^	3.36^(3)^	15.7	6.4
*C67 (3)*	3.99^(1)^	3.21^(4)^	49.6	3.4
*C81 (4)*	3.27^(11)^	3.49^(2)^	8.8	3.2
*C207 (5)*	3.13^(14)^	3.12^(9)^	14.6	13
*C65 (6)*	3.65^(5)^	3.15^(6)^	2.4	2.2
*C148*	3.47^(7)^	–	16.7	10.3
*C980*	–	3.12^(8)^	13	5.8
*C186*	n.a.^e^	3.18^(5)^	n.a.^e^	345.4
*C198*	3.22^(12)^	3.10^(10)^	63.7	18.4
*C200*	3.31^(9)^	3.14^(7)^	5.8	3.4
*C21*	3.09^(15)^	–	2.3	0.9
*C2605*	3.29^(10)^	–	162.7	172.8
C15	3.76^(3)^	–	1.3	1.0
C43	3.49^(6)^	–	1.8	0.8
C58	3.15^(13)^	–	1.4	1.1
C2133	3.39^(8)^	–	1.7	0.9
***Molinia* phylotypes**				
*M7 (1)*	3.18^(9)^	3.37^(2)^	2.6	4.9
M44 (2)	3.63^(5)^	3.37^(1)^	1.9	1.5
*M150 (3)*	3.27^(7)^	–	7.4	4.2
*M71 (4)*	3.73^(4)^	–	120.0	7.5
*M77 (5)*	3.59^(6)^	3.15^(4)^	2.5	2.1
M39 (6)	3.78^(3)^	–	1.6	1.6
*M78*	3.80^(2)^	–	51.0	84.3
*M106*	3.94^(1)^	3.35^(3)^	3.5	3.6
M33	3.19^(8)^	–	1.7	1.7
M55	–	3.14^(5)^	1.0	1.2

^a^Listed are phylotypes that fulfilled the following criteria of LEfSe analyses (Segata et al., 2011): significantly (*P* ≤ 0.05; Kruskal–Wallis test) higher relative abundances in H_2_ supplemented soil treatments compared to unsupplemented soil treatments and effect sizes (LDA-scores) of ≥3. Phylotypes printed in italics had relative abundance ratios [RA ratios; calculated by dividing mean relative abundances of H_2_ supplemented soil treatments (SHC and SHM) by those of unsuplemented soil treatments (SUC and SUM)] of ≥2; these phylotypes were considered as “stimulated by H_2_.”

^b^PT, phylotype; S, shared phylotypes (i.e., *Carex* phylotypes that shared 100% 16S rRNA gene sequence similarity with *Molinia* phylotypes; see Figure 2).

^c^Numbers in parentheses display the rank in the LEfSe-Linear discriminant analyses. –, the phylotype had a LDA score of <3 or had a *P* value of >0.05 in the Kruskal–Wallis test.

^d^Analyses were based on relative abundances of 16S rRNA and 16S rRNA genes, respectively.

^e^n.a., no 16S rRNA sequence of phylotype C186 was detected in Treatment SUC.

**FIGURE 3:**
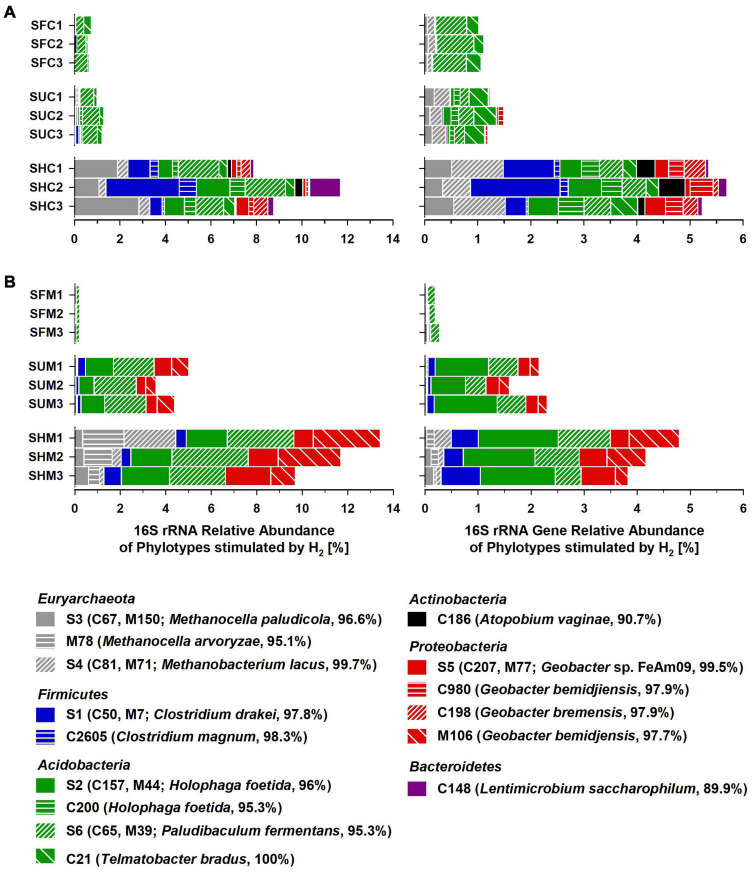
Relative abundance of phylotypes stimulated by supplemental H_2_ in *Carex* **(A)** and *Molinia* **(B)** soil treatments. Displayed phylotypes were considered as “stimulated by H_2_” based on a manually refined LEfSe approach (Table 2). Samples/Treatments: SFC, fresh *Carex* soil; SUC, unsupplemented *Carex* soil; SHC, H_2_ supplemented *Carex* soil; SFM, fresh *Molinia* soil; SUM, unsupplemented *Molinia* soil; SHM, H_2_ supplemented *Molinia* soil; numbers specify replicates. Phylotype identifiers: C/M, phylotypes from experiments with *Carex*/*Molinia* soil; S, shared phylotypes (i.e., *Carex* phylotypes that shared 100% 16S rRNA gene sequence similarity with *Molinia* phylotypes; Figure 2); closest cultured relatives and BLASTn identities are given in parentheses.

The phylotypes stimulated by H_2_ collectively accounted for 7.9–13.4% of the 16S rRNA sequences and 3.8–5.7% of the 16S rRNA gene sequences in Treatment SHC or SHM, which was higher than in unsupplemented soil (Treatments SUC and SUM) and fresh soil (SFC and SFM; Figure 3). Some of these phylotypes were phylogenetically affiliated with acetogenic *Clostridium* species, methanogenic *Euryarchaeota*, and iron reducers of the genus *Geobacter*. Other phylotypes fell within the physiologically diverse phylum *Acidobacteria* or were only distantly related to any cultured organism (Figure 2). In Table 3, potential ecological functions of the phylotypes stimulated by H_2_ were discussed based on physiological traits and genomic potentials of cultured relatives.

**TABLE 3:** Description of phylotypes stimulated by H_2_ in soil treatments ^a^.

PTs	Description
S1, C2605	S1 (97.8% Id. to *Clostridium drakei*) and C2605 (98.3 %Id. to *C. magnum*) phylogenetically clustered within a subgroup of H_2_-utilizing acetogens of the genus *Clostridium* that also harbors non-acetogenic fermenters.^1,2^ The relatedness to H_2_-utilizing acetogens and the finding that S1 and C2605 were stimulated by supplemental H_2_ in soil treatments support the assumption that both PTs represent fen acetogens capable of hydrogenotrophic growth. Acetogens of the genus *Clostridium* do not rely on H_2_ and can grow on various other substrates like sugars, organic acids, and alcohols.^3,4^
S2, C200	S2 and C200 were affiliated to the *Holophagaceae*, a family within the subdivision eight of the phylum *Acidobacteria*, and the acetogen *Holophaga foetida* was the closest cultured relative (96.0% Id. to S2 and 95.3% Id. to C200).^5,6^ Although *H. foetida* does not utilize H_2_, S2, and C200 were stimulated by supplemental H_2_, indicating that the ability to utilize H_2_ may differ between the fen PTs and the cultured reference organism. In this regard, *Holophaga*-affiliated PTs were detected in formate treatments of fen Schlöppnerbrunnen soil, in which formate-derived H_2_ was readily available and acetogenesis was a prominent process; thus, current and previous findings support that the study site harbors H_2_-utilizing *Holophaga*-affiliated acetogens.^7^ Nevertheless, the acetogenic nature of *Holophaga*-affiliated fen PTs like S2 and C200 still needs to be confirmed, preferentially by isolation and subsequent genomic and physiological characterization.
S3, M78	S3 (96.6% Id. to *Methanocella paludicola*) and M78 (95.1% Id. to *M. arvoryzae*) affiliated to the *Methanocellales* (“Rice Cluster 1”), an order currently comprising three validly published species which are all hydrogenotrophic methanogens from paddy rice field soils.^8,9,10^ An additional *Methanocella* sp., K-5A2, could be highly enriched (but not yet isolated) from a *Sphagnum* peat bog and this hydrogenotrophic methanogen was closely related to S3 (99% Id.).^11^ *Methanocella*-affiliated PTs were detected in fresh peat soil of the fen Schlöppnerbrunnen and in anoxically incubated peat soil of this site, either unsupplemented or supplemented with various substrates (e.g., cellulose, xylose, glucose, ethanol, butyrate, propionate, formate, H_2_-CO_2_, and CO_2_).^7,12,13,14,15^ That S3 and M78 were stimulated by supplemental H_2_ in soil treatments underscored recent assumptions that uncultured *Methanocella*-affiliated methanogens are important H_2_ consumers in the study site.
S4	The stimulation of S4 by supplemental H_2_ was in line with the physiological properties of its closest cultured relative, *Methanobacterium lacus* AL-21 (99.7% Id.), a hydrogenotrophic methanogen isolated from a fen in Alaska.^16^ *Methanobacterium* sp. have been isolated from several peatlands thus far, but their importance *in situ* is unknown.^16,17,18^ Previous studies with fresh or incubated peat soil of the fen Schlöppnerbrunnen showed that relative abundances of *Methanobacterium* were lower than those of other hydrogenotrophic methanogens (e.g., *Methanoregula* and *Methanocella*).^7,13,14,15^
S5, C980, C198, M106	S5 was closely related to *Geobacter* sp. FeAm09 (99.5% Id.), while *G. bremensis* and *G. bemidjensis* were the closest cultured relatives (97.7–97.9% Id.) of C980, C198, and M106. These reference organisms are metabolically versatile iron reducers that have been shown to utilize H_2_ (*G. bremensis* or *G. bemidjensis*) or have the genomic potential for H_2_ oxidation (*Geobacter* sp. FeAm09).^19,20,21,22^ Thus, it is possible that *Geobacter*-affiliated fen PTs thrived on supplemental H_2_ and endogenous ferric iron in soil treatments. In addition, these PTs might have consumed acetate, an assumption supported by the ability of acetate oxidation reported for the reference organisms. PTs related to *G.* sp. FeAm09, *G. bremensis* and *G. bemidjensis* have been previously detected in enrichments of H_2_ or acetate utilizing iron reducers from another fen in the same catchment as the study site.^23^
S6	The closest cultured relative of S6 (95.3 % Id.) was the peat acidobacterium *Paludibaculum fermentans* (subdivision 3 of the *Acidobacteria*), a sugar-utilizing facultative aerobe that can ferment and reduce ferric iron in the absence of O_2_ (its capability to utilize H_2_ was not tested).^24^ Considering the low identity, the physiological traits of S6 and *P. fermentans* may differ. Nevertheless, the phylogenetic affiliation with an iron reducer and the finding that a phylotype closely related to S6 was detected in formate treatments of fen Schlöppnerbrunnen soil, in which H_2_ levels were elevated, support the assumption that S6 may represent a H_2_-utilizing iron reducer.^7^ This assumption still needs verification.
C21	C21 showed 100 % 16S rRNA sequence identity to *Telmatobacter bradus* (subdivision 1 of the *Acidobacteria*). *T. bradus* is a facultative aerobe that can ferment sugars and polysaccharides (including cellulose) to H_2_, CO_2_, acetate, and ethanol.^25^ Whether *T. bradus* can utilize H_2_ and reduce alternative electron acceptors like ferric iron or sulfate has not been tested yet. Based on metaomic analyses it was proposed that some subdivision 1 *Acidobacteria* of the fen Schlöppnerbrunnen might couple H_2_-oxidation to sulfate reduction.^26^ In addition, some isolates of subdivision 1 can reduce ferric iron.^27^ Nevertheless, it remains unresolved why the *Telmatobacter*-PT C21 was stimulated by H_2_ in the conducted soil treatments.
C186, C148	C186 and C148 were only distantly related (Id. <91%) to any cultured microbes. Closely related PTs have been detected in the fen before, but their physiologies remain unknown.

^a^References: ^1^, (Gößner et al., 2008); ^2^, (Bomar et al., 1991); ^3^, (Liou et al., 2005); ^4^, (Drake et al., 2008); ^5^, (Bak et al., 1992); ^6^, (Liesack et al., 1994); ^7^, (Hunger et al., 2011); ^8^, (Sakai et al., 2008); ^9^, (Sakai et al., 2010); ^10^, (Lü and Lu, 2012); ^11^, (Sizova et al., 2003); ^12^, (Hamberger et al., 2008); ^13^, (Hunger et al., 2015); ^14^, (Schmidt et al., 2015); ^15^, (Schmidt et al., 2016); ^16^, (Cadillo-Quiroz et al., 2014); ^17^, (Zellner et al., 1988); ^18^, (Kotsyurbenko et al., 2007); ^19^, (Straub and Buchholz-Cleven, 2001); ^20^, (Nevin et al., 2005); ^21^, (Aklujkar et al., 2010); ^22^, (Yadav et al., 2021); ^23^, (Küsel et al., 2008); ^24^, (Kulichevskaya et al., 2014); ^25^, (Pankratov et al., 2012); ^26^, (Hausmann et al., 2018); and ^27^, (Blöthe et al., 2008). PT, phylotype.

In summary, the manually refined LEfSe approach conducted in this study was appropriate to reveal phylotypes stimulated by H_2_ in soil treatments. Some of these phylotypes were related either to hydrogenotrophic acetogens or hydrogenotrophic methanogens and these phylotypes might have contributed to the observed accumulation of acetate and CH_4_, respectively, (Figure 1A). Other phylotypes presumably represent iron reducers and might have been involved in early consumption of exogenous H_2_ (Table 1); alternatively, they were stimulated by acetate derived from hydrogenotrophic acetogenesis.

### Anaerobic processes driven by peat organic carbon in soil treatments

**FIGURE 4:**
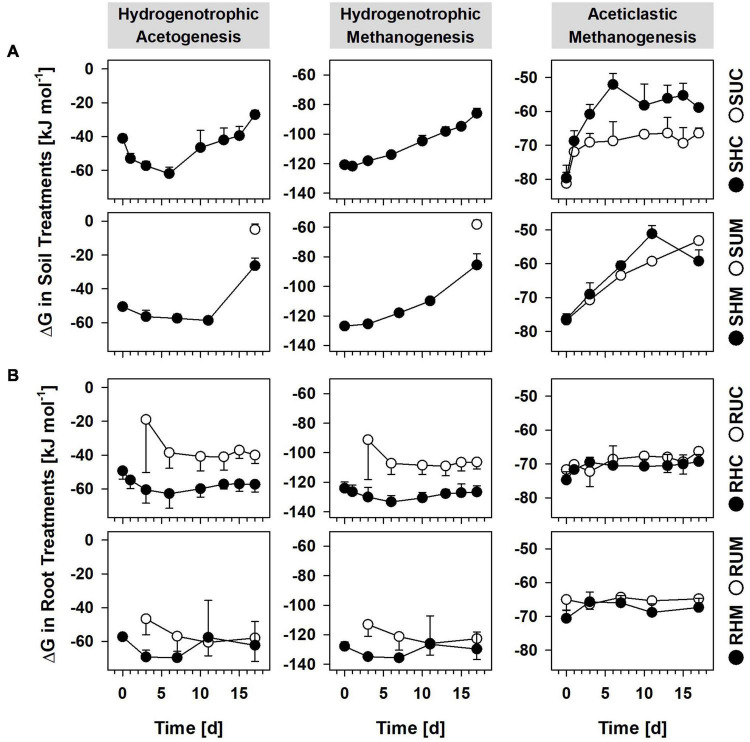
Gibbs free energies (ΔG) of anaerobic processes in soil treatments **(A)** and root treatments **(B)**. ΔGs were calculated based on the following reactions: hydrogenotrophic acetogenesis, 4H_2_ + 2CO_2_ → CH_3_COO^–^ + H^+^ + 2H_2_O; hydrogenotrophic methanogenesis, 4H_2_ + CO_2_ → CH_4_ + 2H_2_O; aceticlastic methanogenesis, CH_3_COO^–^ + H^+^ → CH_4_ + CO_2_. •, H_2_ treatments; °, unsupplemented treatments; when H_2_ was below the detection limit (∼10 ppmV) no ΔGs could be calculated for hydrogenotrophic acetogenesis and hydrogenotrophic methanogenesis. Values represent means of triplicate analysis and error bars indicate standard deviations. Treatments: SHC, H_2_ supplemented *Carex* soil; SUC, unsupplemented *Carex* soil; SHM, H_2_ supplemented *Molinia* soil; SUM, unsupplemented *Molinia* soil; RHC, H_2_ supplemented *Carex* roots; RUC, unsupplemented *Carex* roots; RHM, H_2_ supplemented *Molinia* roots; and RUM, unsupplemented *Molinia* roots.

H_2_ partial pressures in unsupplemented soil treatments were mostly below the detection limit of ∼10 ppmV. Hence, the calculation of Gibbs free energies (ΔGs) for hydrogenotrophic acetogenesis and hydrogenotrophic methanogenesis was not possible. However, solely CO_2_ but neither acetate nor CH_4_ accumulated in treatments SUC and SUM (Figure 1A), suggesting that, in the absence of root organic carbon, respiratory anaerobes thriving on alternative electron acceptors like ferric iron or sulfate were involved in the mineralization of organic carbon and outcompeted acetogens and methanogens for endogenous H_2_. This assumption is supported by long lag phases for methane accumulation and immediate iron reduction in unsupplemented bulk peat soil incubations from the study site (Reiche et al., 2008). Furthermore, thermodynamic calculations indicated that *in situ* iron and sulfate reducers can outcompete acetogens and methanogens for dissolved H_2_ in bulk peat soil of fen Schlöppnerbrunnen (Knorr et al., 2009; Estop-Aragonés et al., 2013). Notably, 6–12 Pa H_2_ were detected at day 17 in Treatment SUM and at this time hydrogenotrophic methanogenesis was sufficiently exergonic (Figure 4A) and CH_4_ concentrations finally increased (Figure 1A). At the same time the mean ΔG for hydrogenotrophic acetogenesis was −5 kJ mol^–1^, a value that has been shown to be the thermodynamic limit of the acetogen *Acetobacterium carbinolicum* (Conrad and Wetter, 1990). In the aforementioned study *A. carbinolicum* had H_2_ thresholds of 10 Pa at 15°C (the incubation temperature in the present study). Thus, it cannot be excluded that H_2_-consuming acetogens contributed to the slight acetate accumulation observed in Treatment SUM at day 17 (Figure 1A).

### Consumers of supplemental H_2_ in soil treatments

In the initial incubation phase in H_2_ supplemented soil treatments, consumption of exogenous H_2_ was not coupled to the accumulation of acetate and CH_4_ (Figure 1A and Table 1), pointing toward the activity of anaerobic H_2_ consumers using electron acceptors others than CO_2_. In this regard, consumption of exogenous H_2_ (or formate) decoupled from methanogenesis and acetogenesis has been reported for soil incubations of fen Schlöppnerbrunnen before (Reiche et al., 2008; Hädrich et al., 2012; Hausmann et al., 2016). Nitrate, ferric iron, sulfate, and humic substances are alternative electron acceptors available in this fen (Estop-Aragonés et al., 2013). Since reported nitrate concentrations are low (0–150 μM) in the fen Schlöppnerbrunnen, it is unlikely that denitrification, a process that is proposed to be important *in situ* (Palmer et al., 2010, 2016), accounted for much of the consumed H_2_ in soil treatments. In contrast, the cumulative electron acceptor capacity of ferric iron, sulfate, and, potentially, humic substances might suffice to account for the unresolved sink of H_2_ in the incubated fen soil (Reiche et al., 2008; Knorr and Blodau, 2009; Knorr et al., 2009; Pester et al., 2012; Estop-Aragonés et al., 2013). In this regard, H_2_ consumption coupled to the reduction of ferric iron is supported by the finding that four phylotypes (S5, C980, C198, and M106) that were stimulated by supplemental H_2_ were related (97.7–99.5% identity) to H_2_-consuming iron reducers of the genus *Geobacter* (Figure 3 and Table 3). In addition, two phylotypes (S6 and C21) were related to *Acidobacteria* that might play a role in H_2_ consumption coupled to the reduction of ferric iron or sulfate (Figure 3 and Table 3). However, only few isolates of the *Acidobacteria* have been physiologically characterized thus far, and potential capabilities to reduce ferric iron or sulfate among the *Acidobacteria* have been largely inferred from genomic data and await validation (Ward et al., 2009; Hausmann et al., 2018).

Subsequently, acetate and CH_4_ accumulated in H_2_ supplemented soil treatments, and reductant recoveries indicated that alternative electron acceptors might have been largely depleted and acetogens as well as methanogens were finally main H_2_ consumers during this second incubation phase (Figure 1A and Table 1). That supplemental H_2_ stimulated acetogenesis and methanogenesis in peat soil treatments corroborated previous studies suggesting that, in fen Schlöppnerbrunnen and other peatlands, acetogens and methanogens are limited by H_2_ availability and both are poised to respond to temporarily higher H_2_ concentrations (Kotsyurbenko et al., 1996; Bräuer et al., 2004; Wüst et al., 2009; Hunger et al., 2011, 2015, 2016; Hädrich et al., 2012). Four phylotypes that were stimulated by H_2_ (Figure 3) were related to cultured acetogens (S1 to *Clostridium drakei*, C2605 to *C. magnum*, S2 and C200 to *Holophaga foetida*) and three to hydrogenotrophic methanogens (S3 and M78 to *Methanolcella* species and S4 to *Methanobacterium lacus*; Table 3). However, BLAST identities of *Holophaga*- and *Methanocella*-related phylotypes to cultured relatives were “only” 95.1–96.6%; thus, isolation and physiological characterization are necessary to validate that these fen phylotypes indeed represent acetogens and methanogens.

### Potential acetate consuming processes in soil treatments

Recently, high relative 16S rRNA abundances of *Methanosaeta* and *Methanosarcina* in fresh peat soil from patches covered with *Carex* indicated that, locally, aceticlastic methanogens can make up a considerable fraction of the active prokaryotes in fen Schlöppnerbrunnen (Meier et al., 2021). In this regard, one phylotype (C44) was related to the aceticlastic methanogen *Methanosarcina acetivorans* (98.7% identity) and it accounted for 1.8–2.5% of all 16S rRNA sequences in the H_2_ supplemented *Carex* soil (SHC, Supplementary Figure 4). Thus, it is possible that some of the acetate produced by acetogens thriving on supplemental H_2_ was subsequently converted into CH_4_ in Treatment SHC (Figure 1A). Likewise, acetate is a growth substrate of the iron reducing *Geobacter* species related to phylotypes S5, C980, C198, and M106 (Table 3), and one may speculate that these phylotypes were not exclusively thriving on supplemental H_2_ but consumed acetate as well. This could explain why acetate accumulation was slow or absent at the beginning of incubation in treatments SHC and SHM, although hydrogenotrophic acetogenesis was sufficiently exergonic right from the start (Figure 4A). Consequently, the calculated reductant recoveries for H_2_ supplemented soil treatments likely underestimated the contribution of acetogens to the consumption of supplemental H_2_ due to presumed acetate consumption by iron reducers and aceticlastic methanogens (Table 1). *In situ*, effective removal of acetate would be advantageous for acetogens since their capability to utilize low H_2_ concentrations depends on low acetate concentrations (Schmidt et al., 2016).

Favorable thermodynamics for aceticlastic methanogenesis (<−60 kJ mol^–1^; Figure 4A) and the finding that the *Methanosarcina*-related phylotype C44 accounted for 1.3–5.9% of the 16S rRNA sequences in unsupplemented *Carex* soil (Treatment SUC, Supplementary Figure 4) raised the question why methane accumulation in this treatment was not observed. Efficient CH_4_ removal *via* anaerobic CH_4_ oxidation potentially coupled to the reduction of ferric iron is one possible explanation and was suggested to occur in peatlands before (Smemo and Yavitt, 2007, 2011; Yan et al., 2018).

### Anaerobic processes in root treatments

To evaluate H_2_ consumption at contrasting H_2_ availabilities by root-associated acetogens and methanogens, *Carex* roots and *Molinia* roots were anoxically incubated with and without supplemental H_2_. Product profiles of unsupplemented roots (Treatments RUC and RUM; Figure 1B and Supplementary Figure 2) corroborated recent findings that (1) H_2_ is produced along with ethanol, CO_2_, acetate, butyrate, and propionate during the fermentative degradation of root-derived organic carbon, and (2) the extent of H_2_ accumulation varies between replicates (Meier et al., 2021). Toward the end of the 17-day incubation, H_2_ consumption exceeded H_2_ production in replicates RUC2 and RUM3, whereas H_2_ production and consumption were balanced in RUC1 and RUC3, and H_2_ production exceeded its consumption in RUM1 and RUM2 (Figure 1B). Independent of overall increasing or decreasing H_2_ concentrations, it is likely that acetogens and fermenters were active at the same time. However, the experimental design did not allow to distinguish between acetate production by hydrogenotrophic acetogenesis, organotrophic acetogenesis, and fermentation.

Previously, formate-derived H_2_, which was subsequently consumed, stimulated the production of acetate and methane in *Carex* root treatments (Hunger et al., 2016), and a similar stimulatory effect was anticipated for supplemental H_2_ in the present study. However, H_2_ concentrations were slowly decreasing or stayed constant in most replicates of root treatments with supplemental H_2_, and neither the accumulation of acetate nor that of CH_4_ (note that CH_4_ was marginal in all root treatments) were significantly higher (*P* ≤ 0.05; Wilcoxon) in the H_2_ supplemented roots (Treatments RHC and RHM) compared to unsupplemented roots (Treatments RUC and RUM; Figure 1B). Nevertheless, the strong accumulation of acetate paralleled by a decrease in H_2_, as observed in RHC1 between day 10 and day 15 and in RHM3 between day 7 and day 11 (Figure 1B), were a strong indicator of hydrogenotrophic acetogenesis in these two replicates.

### Acetogenic phylotypes in root treatments

The manually refined LEfSe approach conducted to reveal phylotypes that were stimulated by supplemental H_2_ in soil treatments was not appropriate for the identification of such phylotypes in root treatments (data not shown). In this regard, fermentation of root-derived organic carbon yielded presumably sufficient H_2_ for root-associated H_2_ consumers, largely obliterating the expected stimulative effect of supplemental H_2_ in root treatments (Figure 1B). In an alternative approach, phylotypes with either ≥1% 16S rRNA or ≥0.5% 16S rRNA gene relative abundance in at least one replicate of any of the root treatments (RUC, RUM, RHC, and RHM) were subjected to a BLASTn analysis and the closest cultured relatives were manually screened for potential H_2_ consumers (e.g., acetogens, methanogens, and iron reducers).

This analysis revealed one phylotype [S1; this phylotype was also active in soil treatments (Figure 3)] with 97.8% identity to the peat bog acetogen *Clostridium drakei* FP (Gößner et al., 2008). In RHM3 and RHC1, i.e., the two replicates of root H_2_ treatments in which H_2_ consumption was most obvious (Figure 1B), phylotype S1 accounted for 9.4% and 3.8% of all 16S rRNA sequences, respectively, (Table 4). However, 16S rRNA and 16S rRNA gene relative abundances of S1 were high in Treatment RUM (Table 4), and in replicates of this treatment H_2_ consumption was less obvious (Figure 1B). Thus, the relative abundance of S1 did not in all cases correlate with observable H_2_ consumption.

**TABLE 4:** Relative abundances of potentially acetogenic phylotypes in fresh and anoxically incubated roots ^a^.

PT	Sample or treatment^b^	Relative abundance [%]^c^					
		16S rRNA			16S rRNA genes		
		Rep 1	Rep 2	Rep 3	Rep 1	Rep 2	Rep 3
S1	RFC	0.05	0.02	0.03	0.01	0.02	0.01
	RUC	0.51	0.12	0.01	0.41	0.03	0.003
	RHC	3.74	0.04	0.02	0.94	0.05	0.005
	RFM	0.02	0.12	0.03	0.03	0.05	0.01
	RUM	4.47	7.19	7.87	2.77	6.45	7.35
	RHM	0.77	4.89	9.36	0.55	3.78	3.76
M227	RFM	0.003	0.02	0.000	0.000	0.003	0.000
	RUM	0.04	1.05	0.94	0.02	1.62	1.43
	RHM	0.12	0.04	1.27	0.11	0.06	0.74

^a^Listed are Phylotypes (PT) with either ≥1% 16S rRNA or ≥0.5% 16S rRNA gene relative abundance in at least one replicate of any root treatment that were closely related to cultured acetogens: S1 with 97.8% identity to *Clostridium drakei* and M227 with 98.3% identity to *Clostridium magnum*.

^b^Identifiers: RFC, fresh *Carex* roots; RFM, fresh *Molinia* roots; RUC, unsupplemented *Carex* roots; RUM, unsupplemented *Molinia* roots; RHC, H_2_ supplemented *Carex* roots; and RHM, H_2_ supplemented *Molinia* roots.

^c^Rep, replicates of fresh root samples or anoxic root treatments.

A second potentially acetogenic phylotype [M227; 98.3% identity to the hydrogenotrophic acetogen *Clostridium magnum* (Bomar et al., 1991)] was observed in *Molinia* root treatments (Table 4). Collectively, S1 and M227, which were marginal in fresh *Molinia* root samples, accounted for up to 10.6% of the 16S rRNA sequences (in RHM3) and 8.8% of the 16S rRNA gene sequences (in RUM3), indicating that root-associated acetogens thrived in *Molinia* root treatments with and without supplemental H_2_ (Table 4).

Phylotypes S1 and M227 were 100% similar to G2 (acc. no. LR702023) and G1 (LR702022), respectively, and the latter two phylotypes were detected in unsupplemented *Carex* and *Molinia* root treatments before (Meier et al., 2021). Furthermore, S1 was 100% similar to an acetogen (LT009683; Figure 2) present in an acetogenic enrichment (FH) that was derived from a mixture of *Carex* and *Molinia* roots collected at fen Schlöppnerbrunnen; enrichment FH converted formate and H_2_ to acetate (Hunger et al., 2016). That phylotypes related to *C. drakei* (S1) and *C. magnum* (M227) were repeatedly observed in treatments with *Carex* and *Molinia* roots suggests that clostridial acetogens colonize the roots of these graminoids in fen Schlöppnerbrunnen.

### Acetogenesis and methanogenesis in root treatments

Thermodynamic calculations revealed that hydrogenotrophic acetogenesis was feasible in unsupplemented roots (Treatments RUC and RUM; Figure 4B) as soon as sufficient fermentation-derived H_2_ accumulated (after 3 to 6 days; Figure 1B). Furthermore, supplemental H_2_ did neither significantly stimulate acetate production (Figure 1B) nor were 16S rRNA and 16S rRNA gene relative abundances of potentially acetogenic phylotypes significantly higher in root treatments with supplemental H_2_ than in those without (Table 4). These findings indicated that acetogens in root treatments did not relay on supplemental H_2_. Nevertheless, H_2_ consumption in root treatments was most obvious in two replicates that received supplemental H_2_ (RHC1 and RHM3; Figure 1B). In RHM3 between day 7 and 11, concentrations of H_2_ and CO_2_ decreased by 6 mM and 2.8 mM, respectively, and acetate increased by 12.8 mM. However, only 1.5 mM acetate can be formed from 6 mM H_2_ *via* hydrogenotrophic acetogenesis. This discrepancy can be explained as follows: (1) A part of the accumulated acetate was presumably produced by fermenters thriving on root-derived organic carbon (Meier et al., 2021). (2) Only net consumption/production of H_2_, CO_2_, and acetate could be determined, but fermentative production of H_2_ and CO_2_ were most likely ongoing concomitantly to their consumption by acetogens in all root treatments. In this regard, radiotracer experiments indicated that 30–40% of acetate in rice root treatments originated from H_2_-CO_2_, although net consumption of H_2_ could only account for 4% of accumulated acetate, pointing toward a fast turnover of H_2_ in these rice root treatments (Conrad and Klose, 1999). (3) The metabolic versatility of acetogens is widely recognized (Drake et al., 2008), and, in the root treatments, acetogens might have utilized root-derived organic carbon (e.g., sugars) and fermentation products [e.g., formate, lactate, and ethanol (Weghoff et al., 2015; Bertsch et al., 2016; Hunger et al., 2016)] in addition to H_2_-CO_2_. In this respect, ethanol concentrations decreased by 4.8 mM in RHM3 between day 7 and 11 (Supplementary Figure 2), and this amount of ethanol can theoretically yield 7.2 mM acetate.

Methanogens can be associated with roots of wetland plants (Kimura et al., 1991; King, 1994; Conrad and Klose, 1999), and, previously, supplemental formate or formate-derived H_2_ stimulated CH_4_ production in treatments with washed *Carex* roots (Hunger et al., 2016). In the present study, CH_4_ accumulation was negligible in root treatments with and without supplemental H_2_ (Figure 1B), although hydrogenotrophic and aceticlastic methanogenesis were highly exergonic (Figure 4B). Since the 16S rRNA and 16S rRNA gene relative abundances of methanogens (and archaea in general) were low (Supplementary Figure 4), it is possible that methanogens were loosely attached to the root surface and were largely removed by the gentle washing procedure. In addition, methanogens may have been inhibited by the high concentrations of organic acids (Figure 1B and Supplementary Figure 2) that at the moderately acidic pH persist largely in their undissociated form and can cause a decoupling of the proton motive force (Luli and Strohl, 1990; Horn et al., 2003).

### Implications for acetogens in the root-zone of fen graminoids

H_2_ was an important product of fermenters thriving on decaying roots and root litter in *Carex* and *Molinia* root treatments conducted in this and previous studies (Hunger et al., 2016; Meier et al., 2021). *in situ*, exudates, constantly released by roots of photosynthetically active plants, are another important source of root-derived organic carbon (Jones et al., 2009), and they might be partially converted to H_2_ as well. H_2_ formed by root-associated fermenters can theoretically (1) be transported through the plants *via* the aerenchyma and emitted to the atmosphere, (2) be consumed by aerobic or anerobic microbial H_2_ consumers colonizing the roots, or (3) radially diffuse into the soil surrounding the roots where it is eventually consumed by soil microbes (Conrad, 1996). Acetogens inhabiting the root zone of graminoids could profit from locally and temporarily higher H_2_ concentrations than the 0.2–28 nmol l^–1^ dissolved H_2_ (corresponds to a H_2_ partial pressure of approximately 0.03–4 Pa) observed in bulk peat soil of the fen Schlöppnerbrunnen (Knorr et al., 2009; Estop-Aragonés et al., 2013). In fact, acetogens have been shown to be associated to the roots of several aquatic plants, and their ability to cope with oxic stress makes them less vulnerable to O_2_ released from the roots than methanogens (Conrad and Klose, 1999; Küsel et al., 1999, 2001; Leaphart et al., 2003; Gößner et al., 2006). As discussed earlier (Conrad and Klose, 1999; Hunger et al., 2016; Meier et al., 2021), the conducted incubations cannot simulate the complex processes ongoing in the root zone of a living plant, but show the potential of its anaerobic microbial community to thrive on supplemental H_2_, fermentation-derived H_2_, and root or peat organic carbon.

**FIGURE 5:**
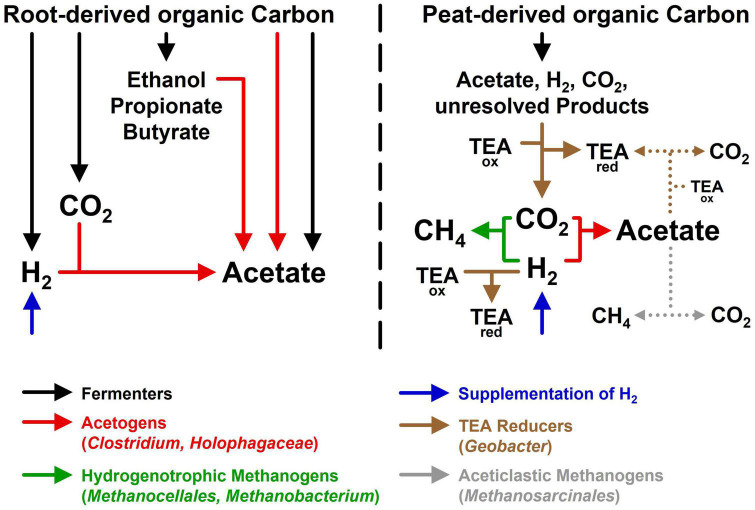
Hypothetical model summarizing the main processes and taxa observed in root treatments (left) and soil treatments (right). TEA_*ox*_, unknown terminal electron acceptors such as ferric iron or sulfate; TEA_*red*_, unknown reduced products (e.g., ferrous iron or sulfide) of anaerobic respiratory prokaryotes. Dotted lines indicate that some of the acetate produced by acetogens may have been consumed by aceticlastic methanogenesis or other acetate-consuming processes.

In Figure 5 the interwoven trophic links in root and soil treatments were graphically summarized to help addressing the initial hypotheses: (1) Phylotypes related to cultured acetogens (*Clostridium* and *Holophaga*) were identified in root treatments and soil treatments, underscoring that acetogens are associated to graminoid roots and inhabit the surrounding peat soil. However, these potential acetogens have yet to be cultured to validate their assumed physiology. (2) Acetogens most likely consumed fermentation derived H_2_ in root treatments and supplemental H_2_ in soil treatments, suggesting that H_2_, temporarily formed in excess at the immediate vicinity of graminoid roots, can be utilized by acetogens, if it accumulates to sufficiently high concentrations. *In situ*, H_2_ will likely diffuse away (Conrad, 1996) and not accumulate to concentrations as high as in root treatments or H_2_ supplemented soil treatments. Therefore, radial H_2_ profiles at high resolution around roots of living graminoids are required to prove that their root zones are indeed microenvironments with higher H_2_ availabilities. Thus far, *in situ* H_2_ concentrations could only be resolved at larger scales at fen Schlöppnerbrunnen (Knorr et al., 2009; Estop-Aragonés et al., 2013). (3) Product profiles in unsupplemented soil treatments corroborated the assumption that in the absence of root-derived organic carbon, acetogens are outcompeted for endogenous H_2_ by H_2_ consumers with lower thresholds. In the iron rich fen Schlöppnerbrunnen, not only hydrogenotrophic methanogens (e.g., *Methanocellales* and *Methanobacterium*) but also iron reducers (e.g., *Geobacter*) and, presumably, sulfate reducers compete with acetogens for available H_2_ (Reiche et al., 2008; Hausmann et al., 2016). However, acetogens are metabolically flexible and do not rely on H_2_ (Drake et al., 2008; Schuchmann and Müller, 2016). *In situ*, acetogens could thrive on root-derived organic carbon, organic fermentation products, and CO in addition to H_2_. In this respect, acetogens can co-metabolize several energy sources (e.g., H_2_ and formate) at the same time or grow mixotrophically (i.e., H_2_ is used as a lithotrophic energy source and organic compounds are used as heterotrophic carbon source). Furthermore, acetogens are well adapted to changing redox conditions that are characteristic for the root-zone of wetland plants (Conrad, 1996; Brune et al., 2000). Thus, when O_2_ and alternative electron acceptors are temporarily and locally depleted, acetogens might dominate H_2_ oxidation until hydrogenotrophic methanogens reestablish H_2_ concentrations below the threshold of acetogens.

## Conclusion

The root-zones of fen graminoids are hotspots for H_2_-producing fermenters in the fen Schlöppnerbrunnen, and it was hypothesized that acetogens may thrive on H_2_ diffusing into the soil around graminoid roots (Hunger et al., 2016; Meier et al., 2021). In the present study, potential acetogenic phylotypes successfully competed with methanogens in soil and root treatments when H_2_ was available in sufficiently high concentrations. However, acetogens and methanogens were outcompeted, possibly by iron reducers, when H_2_ concentrations were low (unsupplemented root-free soil treatments). To prove that acetogens can indeed thrive on H_2_ in the root-zones of graminoids in the fen Schlöppnerbrunnen and other peatlands, radial H_2_ profiles at high resolution around roots of living plants would be required. Nevertheless, especially those acetogens that are more tightly associated to the roots (e.g., acetogenic phylotypes that were detected in root-treatments; Table 4) could thrive directly on root-derived organic carbon or organic fermentation products (e.g., ethanol) in addition to H_2_.

By shifting the flow of carbon and reductant toward acetate during the anaerobic degradation of root organic carbon, hydrogenotrophic and organotrophic acetogens collectively can limit the H_2_ availability for hydrogenotrophic methanogens (Conrad, 1999; Schuchmann and Müller, 2016). When acetate is subsequently not consumed by aceticlastic methanogens, as observed in a peatland in Alaska (Duddleston et al., 2002), the overall production of CH_4_ is low. Thus, acetogens in the root zone of graminoids could be involved in controlling CH_4_ production in some peatlands, and further studies are needed to better resolve trophic links between acetogens and acetate consuming prokaryotes in these globally relevant ecosystems.

## Data availability statement

The data presented in the study are deposited in the European Nucleotide Archive (ENA) repository, accession numbers: PRJEB37304 and PRJEB37863; the data have been released.

## Author contributions

AM, SO, HD, and OS conceived to the study. AM performed the experiments. AM and OS analyzed the data. OS wrote the manuscript with input from all authors. All authors have read and agreed to the final version of the manuscript.

## Footnotes

1
http://cran.at.r-project.org

2
https://folk.uio.no/ohammer/past/
