# Supplementary material for: The root zone of graminoids: A niche for H2-consuming acetogens in a minerotrophic peatland
Source: Front Microbiol. 2022 Aug 5;13:978296. doi: 10.3389/fmicb.2022.978296 (PMC9391049; doi:10.3389/fmicb.2022.978296)
Supplement: Supplementary file 1 [file Data_Sheet_1.docx]

Supplementary Material

**Supplementary Text**

**Supplementary Text 1** Microbial community composition in soil treatments and root treatments.

**Supplementary Figures**

**Fig. S1** Experimental setup.

**Fig. S2** Ethanol, butyrate, propionate, and pH in soil treatments (A) and root treatments (B).

**Fig. S3** Non-metric multidimensional scaling (NMDS) analyses of *Carex* root and soil samples (A) and *Molinia* root and soil samples (B).

**Fig. S4** Prokaryotic community composition of incubated and unincubated soil and roots.

**Supplementary Tables**

**Table S1** Alpha diversity of 16S rRNA and 16S rRNA gene sequence data sets of fresh and anoxically incubated soil.

**Table S2** Alpha diversity of 16S rRNA and 16S rRNA gene sequence data sets of fresh and anoxically incubated roots.

**Supplementary Text 1**

**Microbial community composition in soil treatments and root treatments**

At the end of the 17-day anoxic incubation 16S rRNA and 16S rRNA gene analyses were performed for all treatments to elucidate metabolically active prokaryotic taxa and the overall prokaryotic community composition, respectively. In addition, these molecular analyses were conducted with fresh, unincubated roots and soil to reveal prokaryotic taxa present and active at the time of sample collection in the fen Schlöppnerbrunnen. 16S rRNA was chosen as molecular marker instead of specific marker (e.g., *mcrA* genes and transcripts for methanogens or *fhs* genes and transcripts for acetogens; (Springer et al., 1995; Leaphart and Lovell, 2001)) to cover the complete prokaryotic community and to reduce the sequencing effort.

Non-metric multidimensional scaling (NMDS) analyses of 16S rRNA and 16S rRNA gene phylotypes (≥ 97% sequence similarity) corroborated recent findings showing that the microbiomes of anoxically incubated *Carex* and *Molinia* roots considerably differ from that of unincubated roots, whereas differences between the microbiomes of fresh root-free soil and anoxically incubated root-free soil were less pronounced (Fig. S3) (Meier et al., 2021). No distinct clustering between prokaryotic communities of H_2_ supplemented roots and unsupplemented roots was observed, and the differences between H_2_ supplemented soil and unsupplemented soil were marginal (Fig. S3).

*Acidobacteria*, *Proteobacteria* and *Actinobacteria* were the dominant prokaryotic phyla in fresh *Carex* soil and fresh *Molinia* soil, and these phyla also dominated in *Carex* and *Molinia* soil treatments with and without H_2_ (Fig. S4). Chao1 and Shannon Diversity indices were also similar between fresh soil, soil treatments with H_2_, and unsupplemented soil treatments of both plants (Table S1).

Alpha diversity parameters were lower in *Carex* root treatments and *Molinia* root treatments compared to fresh *Carex* roots and fresh *Molinia* roots, respectively (Table S2). These results suggest that a subset of the microbes associated to the roots thrived under the experimental conditions. In this regard, the microbial communities of *Carex* root treatments with and without supplemental H_2_ were dominated by *Enterobacteriaceae*, *Clostridiaceae*, *Veillonellaceae*, *Neisseriaceae*, *Acidobacteriaceae* and *Acidothermaceae*, while *Clostridiaceae* dominated in *Molinia* root treatments with and without supplemental H_2_ (Fig. S4). Most of these families were largely represented by a few phylotypes that were recently identified as important fermenters associated with *Carex* and *Molinia* roots (Meier et al., 2021).

Collectively, these results suggested that supplemental H_2_ had a minor effect on the overall microbial community composition in root treatments and soil treatments of both plants, and a more detailed analysis was necessary to identify potential soil-born or root-associated H_2_ consumers.

Leaphart, A. B., and Lovell, C. R. (2001). Recovery and analysis of formyltetrahydrofolate synthetase gene sequences from natural populations of acetogenic bacteria. *Appl. Environ. Microbiol.* 67, 1392–1395. doi: 10.1128/AEM.67.3.1392-1395.2001.

Meier, A. B., Oppermann, S., Drake, H. L., and Schmidt, O. (2021). Organic carbon from graminoid roots as a driver of fermentation in a fen. *FEMS Microbiol. Ecol.* 97, fiab143. doi: 10.1093/femsec/fiab143.

Springer, E., Woese, C. R., and Boone, D. R. (1995). Partial gene sequences for the A subunit of methyl-coenzyme M reductase (mcrI) as a phylogenetic tool for the family methanosarcinaceae. *Int. J. Syst. Bacteriol.* 45, 554–559. doi: 10.1099/00207713-45-3-554.


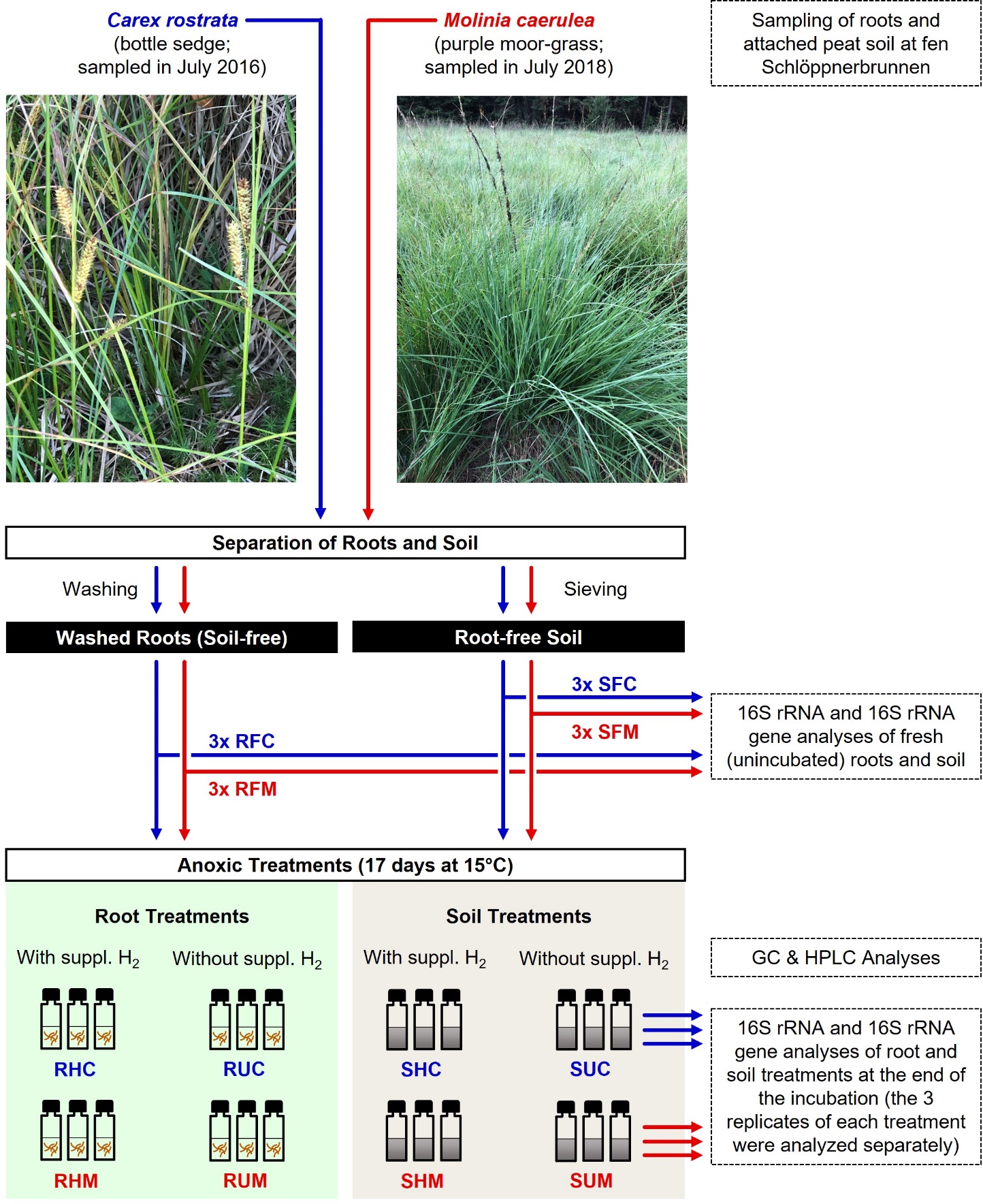


**Fig. S1** **Experimental setup.** Unincubated samples: SFC, fresh *Carex* soil; SFM, fresh *Molinia* soil; RFC, fresh *Carex* roots; RFM, fresh *Molinia* roots. Treatments: SUC, unsupplemented *Carex* soil; SHC, H_2_ supplemented *Carex* soil; SUM, unsupplemented *Molinia* soil; SHM, H_2_ supplemented *Molinia* soil; RUC, unsupplemented *Carex* roots; RHC, H_2_ supplemented *Carex* roots; RUM, unsupplemented *Molinia* roots; RHM, H_2_ supplemented *Molinia* roots.


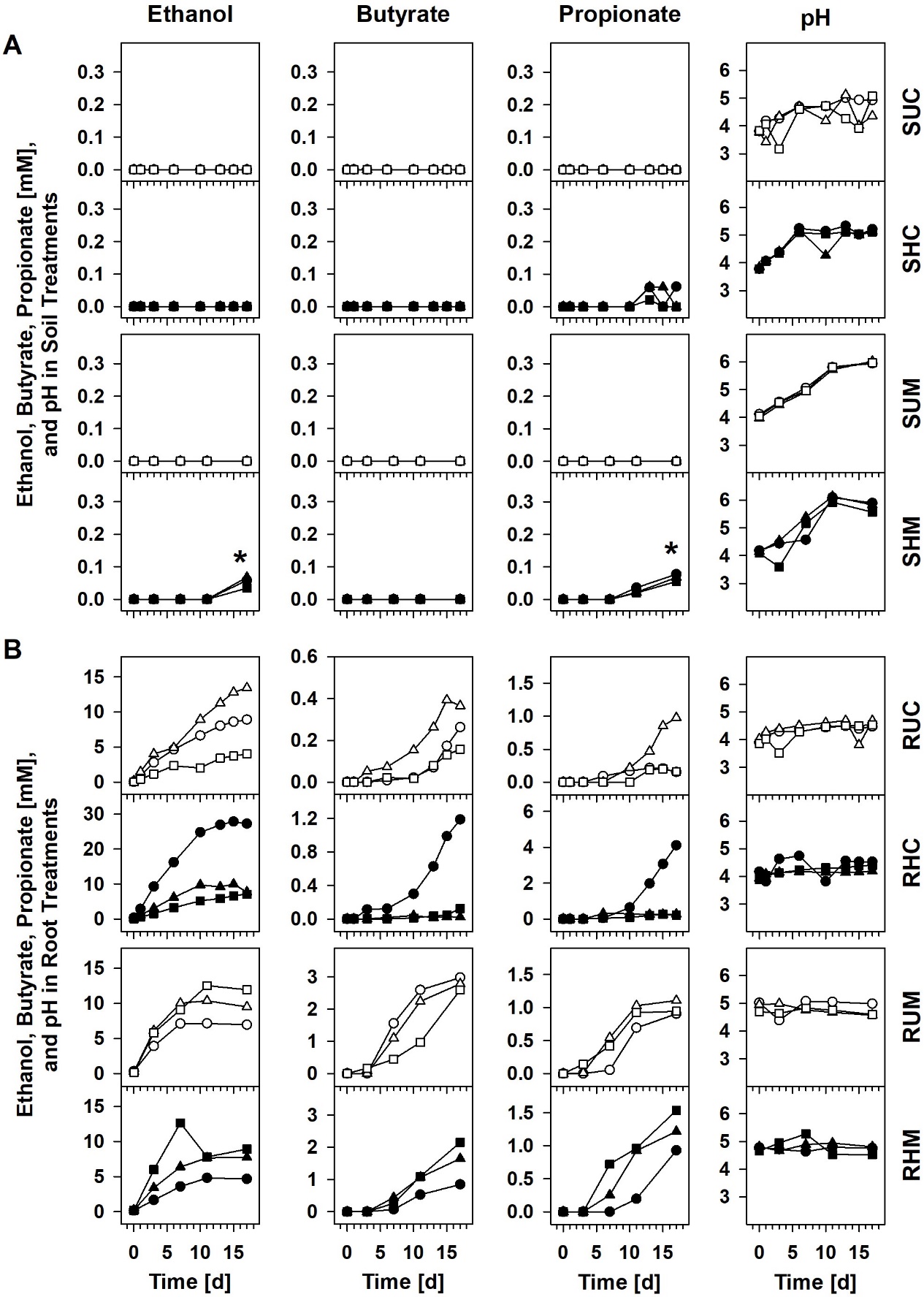


**Fig. S2** **Ethanol, butyrate, propionate, and pH in soil treatments (A) and root treatments (B).** Treatment identifiers: SUC, unsupplemented *Carex* soil; SHC, H_2_ supplemented *Carex* soil; SUM, unsupplemented *Molinia* soil; SHM, H_2_ supplemented *Molinia* soil; RUC, unsupplemented *Carex* roots; RHC, H_2_ supplemented *Carex* roots; RUM, unsupplemented *Molinia* roots; RHM, H_2_ supplemented *Molinia* roots. Symbols: circles, replicate 1; triangles, replicate 2; squares, replicate 3. The asterisks indicate significant differences (one-sided Wilcoxon rank sum test; *P* ≤0.05) between the amounts of ethanol, butyrate, and propionate formed in H_2_ treatments and unsupplemented treatments during incubation. See Fig. 1 for concentrations of H_2_, CO_2_, CH_4_, and acetate.


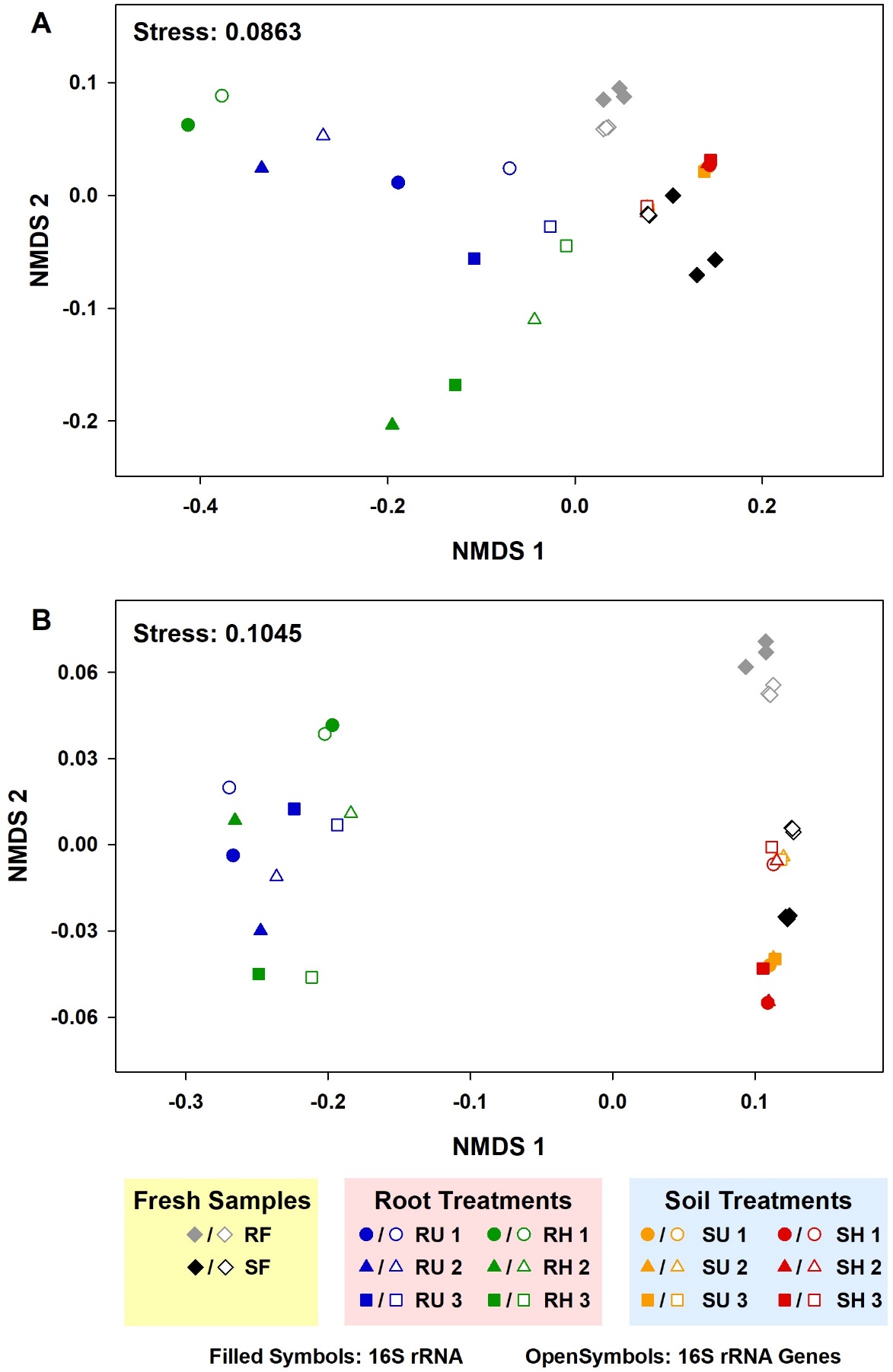


**Fig. S3 Non-metric multidimensional scaling (NMDS) analyses of *Carex* root and soil samples (A) and *Molinia* root and soil samples (B).** Distance matrices (Bray-Curtis) are based on 16S rRNA (filled symbols) and 16S rRNA gene (open symbols) relative abundances of all detected phylotypes (≥ 97 % sequence similarity). Sample identifiers: RF, fresh roots; SF, fresh soil; RU, unsupplemented root treatments; RH, H_2_ supplemented root treatments; SU, unsupplemented soil treatments; SH, H_2_ supplemented soil treatments; Numbers indicated different replicates; See Fig. S1 for the experimental design.


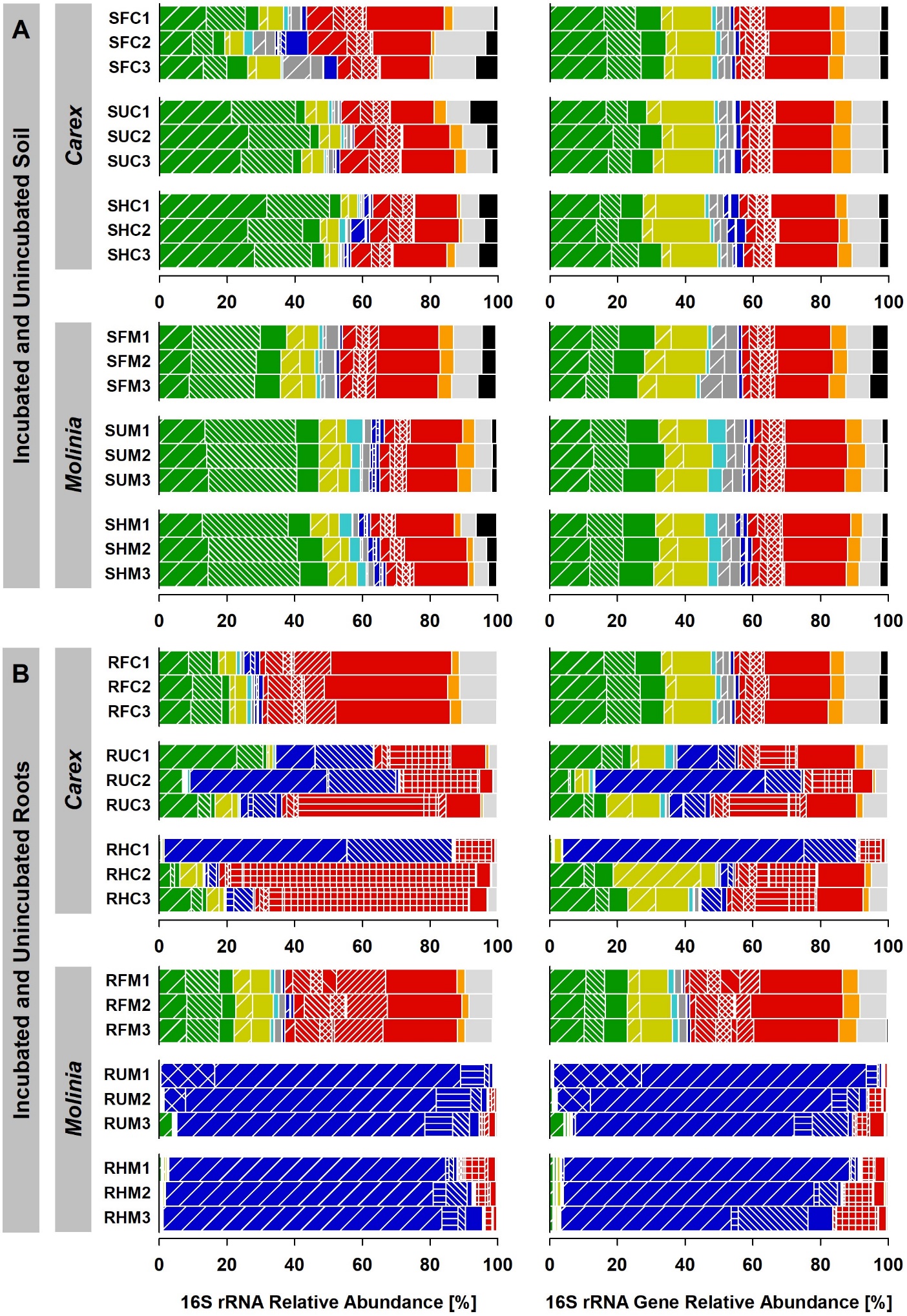


**Fig. S4 Prokaryotic community composition of incubated and unincubated soil and roots.** (continued)


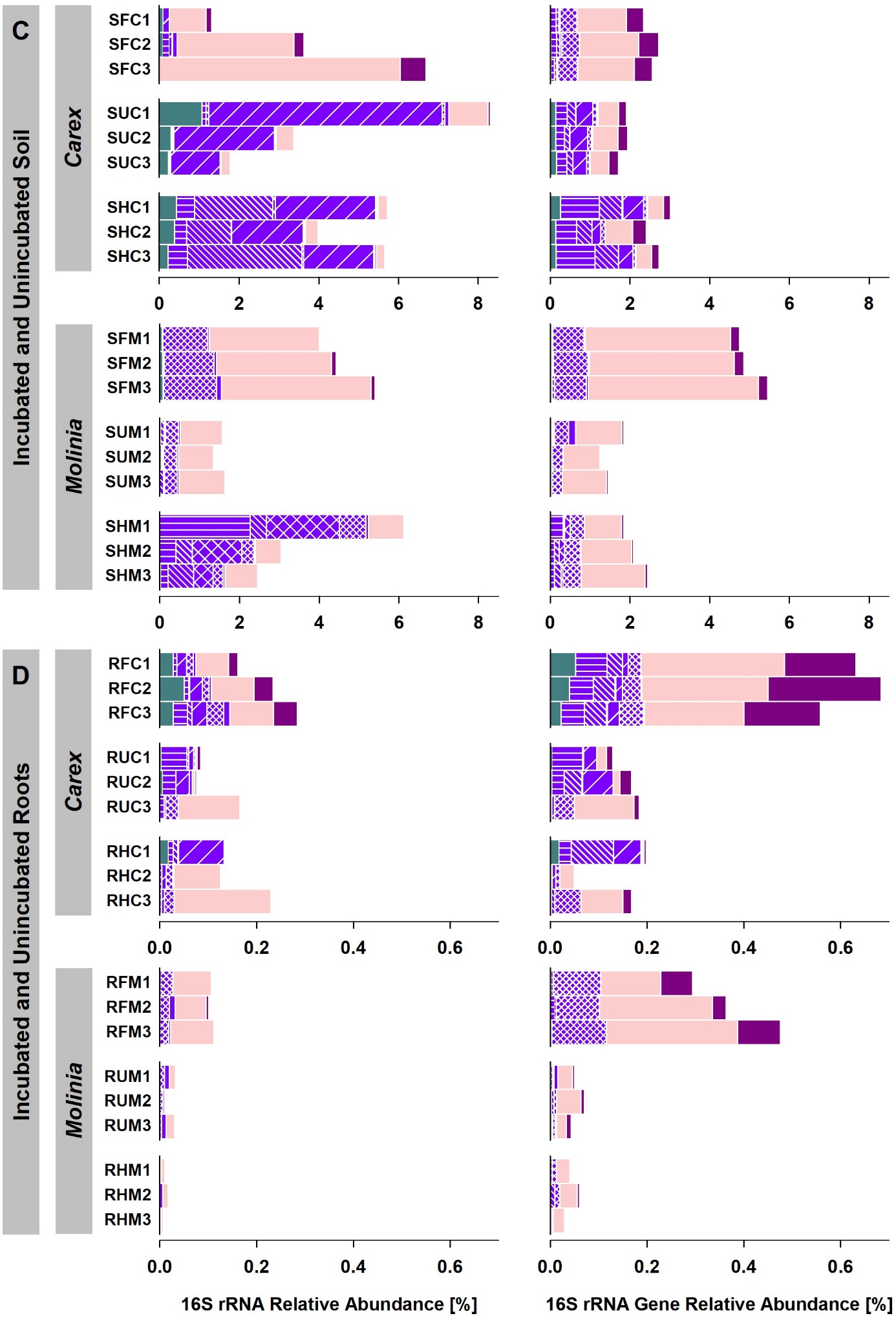


**Fig. S4 Prokaryotic community composition of incubated and unincubated soil and roots.** (continued)


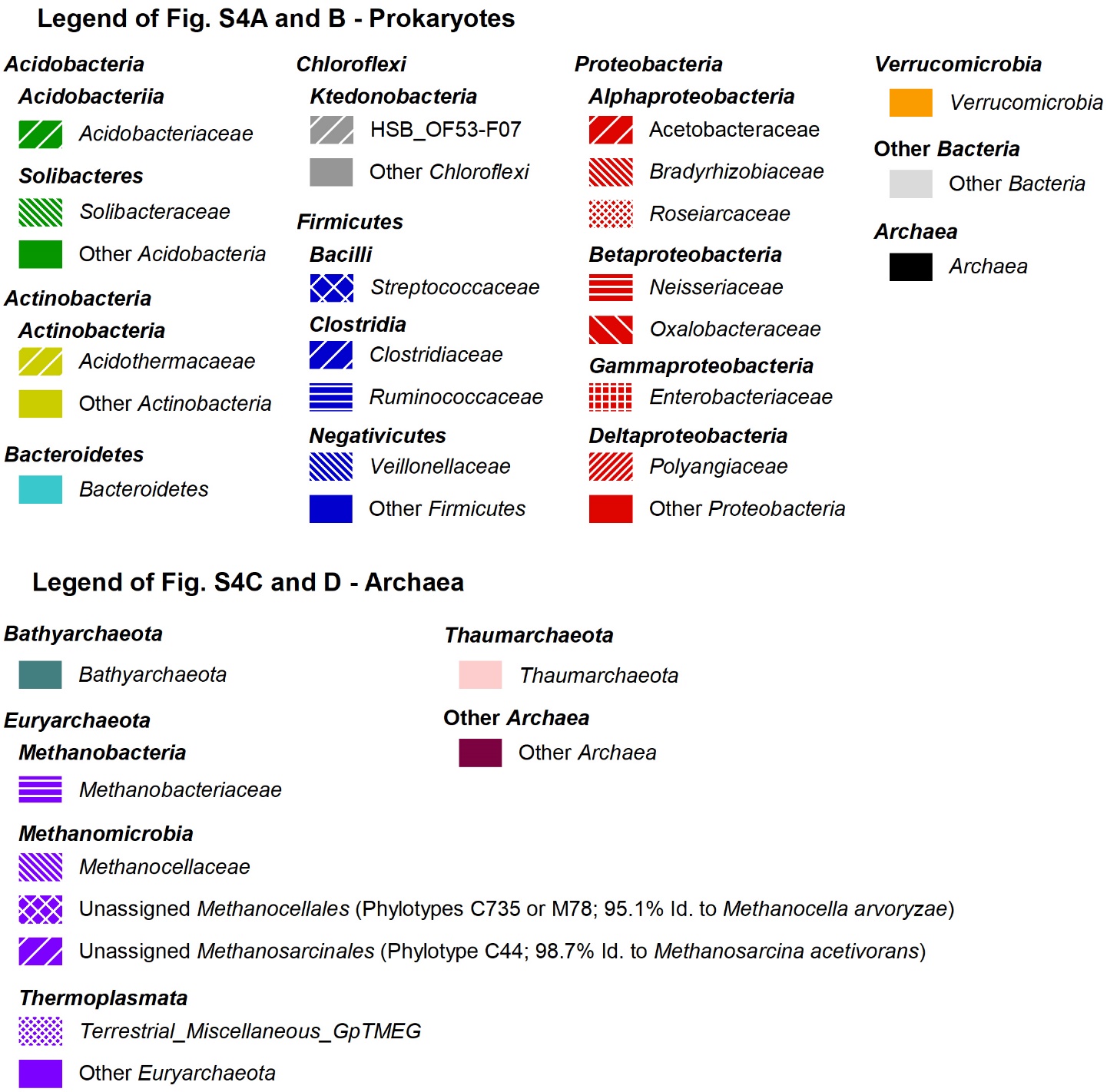


**Fig. S4 Prokaryotic community composition of incubated and unincubated soil and roots.** Panels A and B, prokaryotic taxa in soil and root samples, respectively; Panels C and D, archaeal taxa in soil and root samples, respectively. Shown are the most important families or phyla (i.e., those with ≥ 5% [panels A and B] or ≥ 0.5% [panels C and D] 16S rRNA or 16S rRNA gene relative abundances in at least one sample). Sample identifier: SFC, fresh *Carex* soil; SUC, unsupplemented *Carex* soil; SHC, H_2_ supplemented *Carex* soil; SFM, fresh *Molinia* soil; SUM, unsupplemented *Molinia* soil; SHM, H_2_ supplemented *Molinia* soil; RFC, fresh *Carex* roots; RUC, unsupplemented *Carex* roots; RHC, H_2_ supplemented *Carex* roots; RFM, fresh *Molinia* roots; RUM, unsupplemented *Molinia* roots; RHM, H_2_ supplemented *Molinia* roots; numbers specify replicate nucleic acid extractions of fresh samples or replicates of anoxic treatments. See Fig. S1 for an overview of the experimental setup.

**Table S1: Alpha diversity of 16S rRNA and 16S rRNA gene sequence data sets of fresh and anoxically incubated soil.^a^**

| **Alpha Diversity Parameter** | **16S rRNA** | | | | | | | | | | |  | **16S rRNA Genes** | | | | | | | | | | |
| --- | --- | --- | --- | --- | --- | --- | --- | --- | --- | --- | --- | --- | --- | --- | --- | --- | --- | --- | --- | --- | --- | --- | --- |
|  | **Fresh Soil** | | |  | **Unsupplemented Soil Treatments** | | |  | **H_2_ supplemented Soil Treatments** | | |  | **Fresh Soil** | | |  | **Unsupplemented Soil Treatments** | | |  | **H_2_ supplemented Soil Treatments** | | |
|  | **1** | **2** | **3** |  | **1** | **2** | **3** |  | **1** | **2** | **3** |  | **1** | **2** | **3** |  | **1** | **2** | **3** |  | **1** | **2** | **3** |
| *Carex rostrata* | | | | | | | | | | | | | | | | | | | | | | | |
| No. of Sequences | 39825 | 4412 | 1721 |  | 32758 | 27056 | 31842 |  | 17483 | 31153 | 27173 |  | 41397 | 49239 | 31668 |  | 35188 | 59941 | 52291 |  | 41341 | 28276 | 28949 |
| Observed Phylotypes (normalized)^c^ | 1516 (186) | 631 (171) | 329 (150) |  | 1114 (161) | 1051 (165) | 1129 (164) |  | 790 (153) | 1101 (171) | 1031 (166) |  | 1617 (188) | 1741 (192) | 1510 (191) |  | 1525 (191) | 1807 (201) | 1752 (197) |  | 1582 (203) | 1425 (209) | 1365 (206) |
| Chao1 (normalized)^c^ | 1901 (240) | 989 (219) | 569 (184) |  | 1629 (217) | 1603 (230) | 1688 (227) |  | 1311 (215) | 1556 (232) | 1619 (225) |  | 2046 (246) | 2163 (254) | 1933 (247) |  | 2091 (249) | 2315 (267) | 2124 (250) |  | 1924 (261) | 1769 (265) | 1861 (271) |
| Shannon (normalized)^c^ | 5.4 (4.5) | 5.3 (4.5) | 4.8 (4.3) |  | 4.7 (4.2) | 4.7 (4.2) | 4.9 (4.3) |  | 4.5 (4.1) | 4.8 (4.3) | 4.8 (4.2) |  | 5.4 (4.6) | 5.4 (4.6) | 5.4 (4.6) |  | 5.3 (4.6) | 5.4 (4.7) | 5.5 (4.6) |  | 5.5 (4.7) | 5.5 (4.8) | 5.4 (4.7) |
| *Molinia caerulea* | | | | | | | | | | | | | | | | | | | | | | | |
| No. of Sequences | 20718 | 22298 | 21809 |  | 28459 | 26530 | 30805 |  | 26017 | 32623 | 28858 |  | 39737 | 36372 | 42452 |  | 52928 | 38400 | 50552 |  | 34655 | 52479 | 48465 |
| Observed Phylotypes (normalized)^c^ | 1193 (297) | 1185 (295) | 1236 (299) |  | 1076 (304) | 1031 (300) | 1090 (303) |  | 975 (296) | 993 (294) | 1046 (300) |  | 1411 (294) | 1379 (294) | 1491 (296) |  | 1503 (303) | 1295 (297) | 1467 (305) |  | 1278 (302) | 1409 (300) | 1436 (303) |
| Chao1 (normalized)^c^ | 1648 (305) | 1535 (301) | 1673 (305) |  | 1414 (316) | 1341 (309) | 1441 (314) |  | 1345 (313) | 1372 (303) | 1465 (311) |  | 1864 (302) | 1785 (302) | 1883 (306) |  | 1923 (314) | 1729 (303) | 1924 (315) |  | 1754 (313) | 1817 (310) | 1907 (311) |
| Shannon (normalized)^c^ | 5.5 (4.8) | 5.5 (4.8) | 5.5 (4.8) |  | 4.9 (4.4) | 4.9 (4.4) | 4.9 (4.4) |  | 4.7 (4.3) | 4.7 (4.4) | 4.8 (4.4) |  | 5.4 (4.8) | 5.4 (4.8) | 5.5 (4.8) |  | 5.4 (4.8) | 5.3 (4.8) | 5.4 (4.9) |  | 5.4 (4.9) | 5.4 (4.9) | 5.4 (4.9) |

^a^Numbers in the table header specify replicate nucleic acid extractions of fresh material or replicates of treatments.

^b^Phylotypes are based on 97% sequence similarity. Data sets were normalized to 1,000 (*Carex*) and 10,000 (*Molinia*) sequences for comparisons of amplicon libraries of different sizes within each plant-specific data set.

**Table S2: Alpha diversity of 16S rRNA and 16S rRNA gene sequence data sets of fresh and anoxically incubated roots.^a^**

| **Alpha Diversity Parameter** | **16S rRNA** | | | | | | | | | | |  | **16S rRNA Genes** | | | | | | | | | | |
| --- | --- | --- | --- | --- | --- | --- | --- | --- | --- | --- | --- | --- | --- | --- | --- | --- | --- | --- | --- | --- | --- | --- | --- |
|  | **Fresh Roots** | | |  | **Unsupplemented Root Treatments** | | |  | **H_2_ supplemented Root Treatments** | | |  | **Fresh Roots** | | |  | **Unsupplemented Root Treatments** | | |  | **H_2_ supplemented Root Treatments** | | |
|  | **1** | **2** | **3** |  | **1** | **2** | **3** |  | **1** | **2** | **3** |  | **1** | **2** | **3** |  | **1** | **2** | **3** |  | **1** | **2** | **3** |
| *Carex rostrata* | | | | | | | | | | | | | | | | | | | | | | | |
| No. of Sequences | 96738 | 45224 | 61583 |  | 28482 | 32883 | 23529 |  | 67010 | 44615 | 30460 |  | 49566 | 44871 | 53265 |  | 40343 | 44603 | 35909 |  | 61804 | 49898 | 42689 |
| Observed Phylotypes (normalized)^c^ | 1314 (166) | 1252 (175) | 1411 (172) |  | 634 (112) | 469 (56) | 557 (117) |  | 320 (36) | 626 (80) | 519 (100) |  | 1534 (166) | 1498 (181) | 1581 (182) |  | 1029 (144) | 826 (94) | 939 (157) |  | 500 (45) | 871 (120) | 1045 (155) |
| Chao1 (normalized)^c^ | 1413 (232) | 1561 (233) | 1750 (227) |  | 1093 (146) | 922 (88) | 862 (171) |  | 575 (74) | 900 (114) | 822 (150) |  | 1716 (237) | 1843 (246) | 1900 (252) |  | 1318 (201) | 1223 (151) | 1244 (209) |  | 826 (88) | 1095 (163) | 1353 (209) |
| Shannon (normalized)^c^ | 5.2 (4.0) | 5.2 (4.3) | 5.1 (4.2) |  | 3.7 (3.4) | 2.5 (2.2) | 3.6 (3.2) |  | 2.2 (2.1) | 2.5 (2.2) | 2.7 (2.4) |  | 5.4 (3.7) | 5.5 (4.2) | 5.4 (4.2) |  | 4.6 (3.9) | 3.0 (2.8) | 4.6 (4.1) |  | 2.1 (2.1) | 4.2 (3.7) | 4.7 (4.2) |
| *Molinia caerulea* | | | | | | | | | | | | | | | | | | | | | | | |
| No. of Sequences | 33843 | 34604 | 31367 |  | 30961 | 31249 | 29439 |  | 37926 | 35780 | 44086 |  | 30597 | 31359 | 35110 |  | 37177 | 37934 | 38836 |  | 44153 | 41167 | 36263 |
| Observed Phylotypes (normalized)^c^ | 1137 (281) | 1164 (283) | 1144 (277) |  | 212 (79) | 303 (113) | 337 (132) |  | 426 (164) | 397 (151) | 347 (118) |  | 1246 (285) | 1281 (288) | 1322 (288) |  | 349 (119) | 408 (145) | 591 (196) |  | 552 (188) | 639 (198) | 450 (165) |
| Chao1 (normalized)^c^ | 1475 (294) | 1486 (300) | 1492 (294) |  | 379 (114) | 737 (158) | 620 (176) |  | 712 (210) | 582 (194) | 574 (172) |  | 1689 (297) | 1672 (299) | 1696 (301) |  | 660 (160) | 703 (210) | 950 (245) |  | 799 (242) | 1047 (246) | 755 (213) |
| Shannon (normalized)^c^ | 5.0 (4.4) | 5.1 (4.5) | 5.0 (4.4) |  | 2.0 (2.0) | 2.2 (2.1) | 2.5 (2.4) |  | 1.8 (1.7) | 2.2 (2.1) | 2.0 (1.9) |  | 5.3 (4.5) | 5.4 (4.7) | 5.4 (4.6) |  | 1.9 (1.9) | 2.4 (2.3) | 2.8 (2.6) |  | 1.8 (1.7) | 2.4 (2.3) | 2.5 (2.4) |

^a^Numbers in the table header specify replicate nucleic acid extractions of fresh material or replicates of the treatments.

^b^Phylotypes are based on 97% sequence similarity. Data sets were normalized to 1,000 (*Carex*) and 10,000 (*Molinia*) sequences for comparisons of amplicon libraries of different sizes within each plant-specific data set.
